# Cationic Nanostructures for Vaccines Design

**DOI:** 10.3390/biomimetics5030032

**Published:** 2020-07-07

**Authors:** Ana Maria Carmona-Ribeiro, Yunys Pérez-Betancourt

**Affiliations:** Biocolloids Laboratory, Instituto de Química, Universidade de São Paulo, São Paulo 05508-000, SP, Brazil; y.betancourt@usp.br

**Keywords:** cationic nanoparticles, dioctadecyldimethylammonium bromide, poly (acrylates), biomimetic lipid/polymer nanoparticles, cationic polymer /biocompatible polymer assemblies, cationic adjuvants, cationic lipids, polycation

## Abstract

Subunit vaccines rely on adjuvants carrying one or a few molecular antigens from the pathogen in order to guarantee an improved immune response. However, to be effective, the vaccine formulation usually consists of several components: an antigen carrier, the antigen, a stimulator of cellular immunity such as a Toll-like Receptors (TLRs) ligand, and a stimulator of humoral response such as an inflammasome activator. Most antigens are negatively charged and combine well with oppositely charged adjuvants. This explains the paramount importance of studying a variety of cationic supramolecular assemblies aiming at the optimal activity in vivo associated with adjuvant simplicity, positive charge, nanometric size, and colloidal stability. In this review, we discuss the use of several antigen/adjuvant cationic combinations. The discussion involves antigen assembled to (1) cationic lipids, (2) cationic polymers, (3) cationic lipid/polymer nanostructures, and (4) cationic polymer/biocompatible polymer nanostructures. Some of these cationic assemblies revealed good yet poorly explored perspectives as general adjuvants for vaccine design.

## 1. Introduction

Purified antigens in subunit vaccines usually lack the danger signals of full pathogens, resulting in poor immunogenicity [1]. Adjuvants then become essential components of modern vaccines, enhancing and guiding the immune response against each specific pathogen [2,3,4,5]. The only adjuvants licensed for human use worldwide are the aluminum-based salts like Al(OH)_3_. Their water dispersions consist of polydisperse and large aggregated particles poorly dispersed in water that are positively charged at the pH of water and can combine with negatively charged antigens such as peptides, proteins, nucleic acids, and RNA [6,7,8]. Other cationic adjuvants based on nanoparticles [9,10,11,12], liposomes [13,14,15], cationic bilayer fragments [9,16,17], supported cationic bilayers on polymeric nanoparticles (NPs) [10], silica [18], or cationic polymers on superparamagnetic iron oxide NPs have also been proposed as effective micro- or nanomaterials able to effectively interact with antigens and antigen-presenting cells (APC) [19].

The uptake of antigen/cationic assemblies depends on size [20,21]. Virus-like NPs (20–200 nm mean diameter) are taken up by endocytosis via clathrin-coated vesicles, caveolae, or their independent receptors and are preferentially ingested by dendritic cells (DC) [22]. Bacteria-like microparticles (500–5000 nm diameter) undergo phagocytosis and primary ingestion by macrophages. Vaccines administered as particles in dispersion are internalized efficiently by APC either by endocytosis or phagocytosis or a combination of both mechanisms [23,24]. Particles with diameters below 500 nm, in particular NPs (40–100 nm diameter), are more efficient to promote CD8 and CD4 type 1 T-helper cell responses than the microparticles (diameters above 500 nm). Similarly, to Al(OH)_3_, large particles usually induce good antibody responses from T-helper cells type 2 [23]. Cationic micro- and nanoparticles are effectively taken up both by macrophages and dendritic cells. After electrostatics promotes the binding of cationic particles and assemblies to APCs, subsequent internalization takes place [12,14,21,25]. Antigens of *Mycobacterium tuberculosis* [15,26], *Chlamydia trachomatis* [12], *Neisseria meningitides* [17,27], *Taenia crassiceps* [9,10], and *Mycobacterium leprae* [13] carried by cationic particles, liposomes, or bilayer fragments containing dioctadecyldimethylammonium bromide (DODAB) cationic lipid enhanced the cellular and humoral antigen-specific immune response [15,16,28]. Excellent reviews are available on the use of a variety of cationic lipids such as dimethylaminoethane–carbamoyl–cholesterol (DC-Chol) and derivatives [29], 1,2-dioleoyl-3-trimethylammonium-propane (DOTAP) [30], DODAB [9,16,31], and others to present antigens [32].

In response to pathogens, the innate immune system recognizes the pathogen-associated molecular patterns (PAMPs) by means of the pattern recognition receptors (PRRs) on the surface and in endosomes of APCs [1,33]. As a second line of defense, the adaptive immune system developed by vertebrates consists in memory T and B cells that employ new synthesized antigen-specific receptors able to recognize pathogen-specific antigens when presented by major histocompatibility complexes (MHC) on the surface of an APC. The balance between activity of T or B cells relies on signals provided by the APC (such as co-stimulatory molecules and precise cytokines) in response to the priming by PAMPs [1]. In summary, the co-delivery of antigen and adjuvant to APCs in subunit vaccines results in up-regulation of co-stimulatory molecules essential for adequate T and B cells stimulation [1]. In order to formulate vaccines, a fundamental comprehension of innate and adaptive immune responses is required: the first PAMPS recognition is made via several receptors (innate immunity) leading to the responses able to activate and differentiate T helper cells with possible B cell (antibody-mediated) and CD8 T cell-mediated adaptive immune responses [33].

## 2. Assemblies from Cationic Lipids and Surfactants

Mimicking nature is a powerful approach for developing novel lipid-based devices for drug and vaccine delivery. Cationic biomimetic particles offered a suitable interfacial environment for adsorption, presentation, and targeting of antigens in vivo. Thereby, antigens can effectively be presented by tailored biomimetic particles for development of vaccines over a range of defined and controllable particle sizes [34]. Lipid supramolecular association with particles has been systematically studied on latex, silica, or drug particles over a range of experimental conditions in order to achieve optimal bilayer deposition onto each particle. The difficult step of vesicle disruption, especially for bilayers in the rigid gel state, was circumvented by using previously disrupted charged vesicles, namely charged bilayer fragments or disks (BF). BF, under appropriate conditions of the intervening medium, coalesced around particles for presentation of antigens to the immunological system [35].

Antigen loading in the vaccine can be driven in cationic assemblies by electrostatic attraction between the antigen and oppositely charged moieties of the adjuvant. For example, cationic DODAB bilayers in water dispersions are available as closed microstructures such as vesicles or open, nano-sized bilayer fragments (BF) obtained by ultrasonic disruption from closed vesicles [28,36,37,38,39]. These micro- or nano-structures efficiently combine with serum proteins [40], recombinant heat-shock proteins from micobacteria [13], purified extracts from parasites such as *Taenia crassiceps* [9,10], ovalbumin (OVA) model antigen [16], genetic material such as DNA [41,42,43], mono- or oligonucleotides such as CpG [44,45,46], and other oppositely charged biomolecules, drugs, nanoparticles, surfaces, or biological cells [28,41]. Several good reviews appeared on the use of a variety of cationic lipids and surfactants to formulate vaccines [21,32,47,48,49].

**Figure 1:**
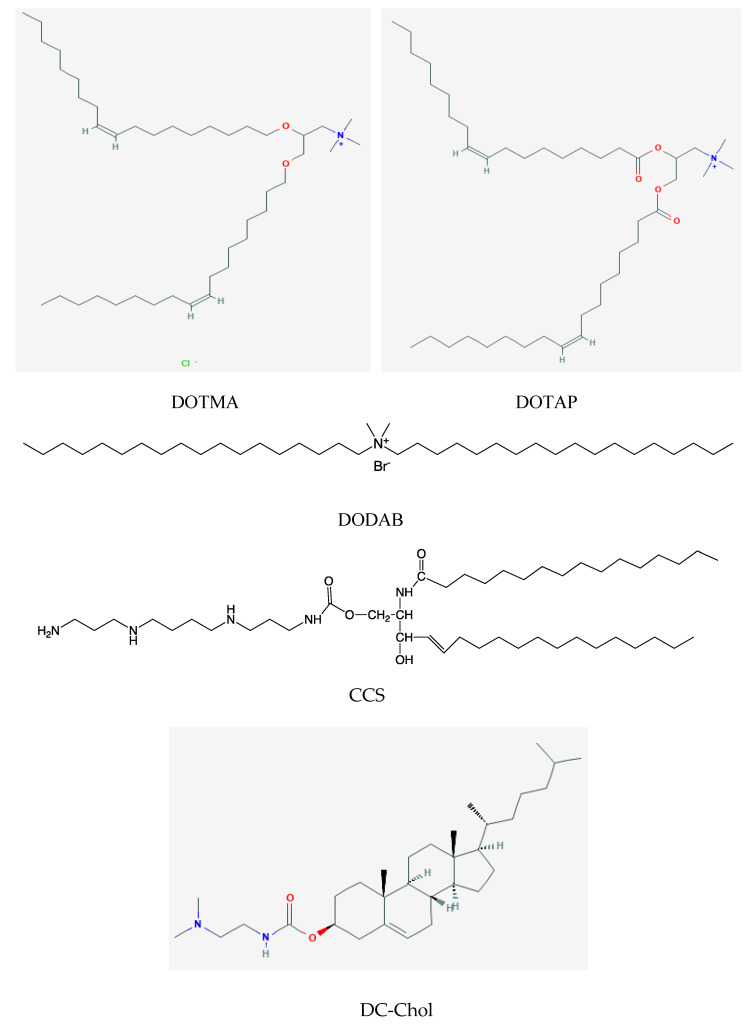
Chemical structure of cationic lipids or surfactants used to formulate vaccines. They bear the cationic charges of primary or secondary amino groups or quaternary ammonium nitrogens in their structures which combine with oppositely charged antigens. CCS is N-palmitoyl d-erythro-sphingosyl-1-0-carbamoyl-spermine triacetate salt. The CCS chemical structure was reprinted from [50] with permission from Elsevier, Copyright 2006.

Examples of monocationic lipids are *N*-(1-(2,3-dioleyloxy)propyl)-*N*,*N*,*N*-trimethylammonium trimethyl chloride (DOTMA), dioleoyl-3-trimethylammonium-propane (chloride salt) (DOTAP), dimethyldioctadecylammonium bromide (DDAB or DODAB), dimethylaminoethane carbamoyl cholesterol (DC-Chol), 1,2-distearoyl-3-trimethylammonium-propane (chloride salt) (DSTAP), and dimyristoyl-3-trimethylammonium-propane (chloride salt) (DMTAP). One example of a polycationic sphingolipid is *N*-palmitoyl d-erythro-sphingosyl-1-0-carbamoyl-spermine triacetate salt (CCS). Figure 1 shows the chemical structures of DOTMA, DOTAP, DODAB, CCS and DC-Chol.

In particular, CCS is a polycationic surfactant with one primary and two secondary amine groups self-assembling as micelles in aqueous phase; CCS required “helper” lipids such as cholesterol to form liposomes before being combined with the hemoaglutinin/neuraminidase antigens of influenza virus to elicit both Th1 and Th2 responses in mice immunized via the nasal route [50,51].

Among the synthetic lipids, DODAB is possibly the less expensive and the most studied synthetic lipid from several points of view such as its self-assembly as closed or open bilayers in aqueous solutions, its physico-chemical properties in aqueous solutions, its ability to interact with several oppositely charged molecules, nanostructures, nanoparticles, surfaces, and cells with a controllable cytotoxicity against mammalian cells lines; its combinations with a large variety of antigens were able to induce a Th1 response but did not improve the Th2 humoral response [9,13,16,28,37,52]. Among the other cationic lipids, only those with more fluid bilayers due to their double bonds in the hydrocarbon chains or to short hydrocarbon chains such as DOTAP or DMTAP, respectively, were able to elicit both Th1 and Th2 responses combined with antigens [50]. The sphingolipid CCS has a large polycationic polar head and inverted-cone molecular shape self-assembling as micelles. Bilayers were formed combining CCS with other neutral lipids such as dioleoyl phosphatidylethanolamine (DOPE) or cholesterol (Chol), at a CCS/helper lipid mole ratio of 1/1 to 4/1; these cationic assemblies were explored by Barenholz and coworkers to deliver influenza virus antigens by the intranasal route [50,51].

The intranasal (i.n.) is an advantageous mucosal route that allows rapid administration for large populations in the case of pandemics. A good example is the i.n. influenza vaccine, based on CCS combining carrier and adjuvant activities, which elicits, in mice, strong systemic (serum) and local (lung and nasal) humoral and cellular immunity. Unsized liposomes of DC-Chol, DODAB, and DSTAP resulted in low serum and local responses, while two others (DMTAP- and DOTAP-based vaccines) induced both systemic and local vigorous Th1 and Th2 immune responses [50]. However, only the vaccine formulated with CCS was equivalent or superior to the commercial vaccine co-administered with cholera toxin as an adjuvant [50]. Innovative mucosal vaccines against influenza [53] or other diseases were recently comprehensively reviewed [54].

**Figure 2:**
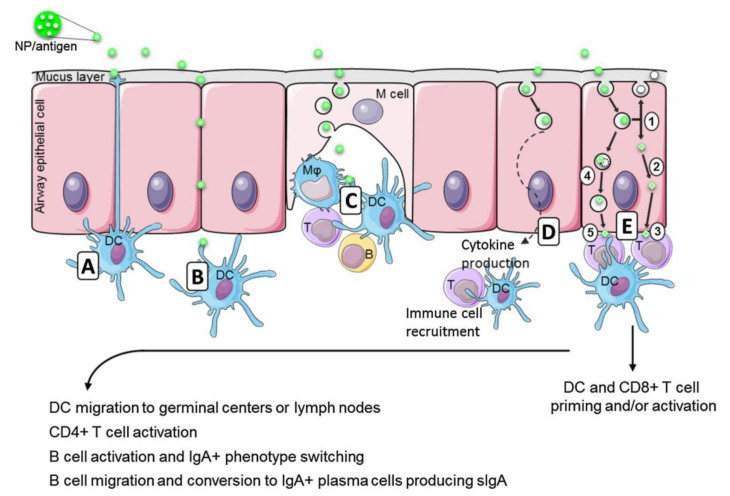
Mucosal immune induction in nasal and airways epithelia by antigen-loaded nanocarriers (NP/antigen). (**A**) Dendritic cells (DC) protruding arms, the transepithelial dendrites, directly capture the NP/antigen. (**B**) NP/antigen can also diffuse through epithelial junctions and reach the underlying DC. (**C**) The M cells create a pocket enriched in immune cells (DC, macrophages-Mφ and lymphocytes T) and perform the sampling of the luminal antigens so that the immune cells contact the NP/antigen. (**D**) The NP/antigen can also enter cells by endocytosis and deliver the antigens of the nanovaccine into the cells. The endocytosis of NP/antigen by the epithelial cells triggers the production of cytokines, defensines, and chemokines involved in local immune cells recruitment (DC and T cells) able to boost the immune response. (**E**) The endocytosis of NP/antigen can also be the first step of antigen presentation by epithelial cells. In endosomes, NP could release antigens and be exocytosed as free, unloaded, NP (E1) and/or induce the endosomal escape of the antigens (E2) that will be processed as an endogenous antigen and presented by major histocompatibility complex I (MHC-I) (E3). In the other way, NP could be degraded in endo/lysosomes (E4), and the released antigen will be processed as exogenous and presented by major histocompatibility complex II (MHC-II) (E5). This could lead to DC and CD8+ T cell activation and/or priming. The activated DC from these pathways then migrate to germinal centers or directly to lymph nodes to activate CD4+ T cells that in turn activate B cells. They undergo an IgA+ phenotype switch, migrate by the blood flow to the effector sites and produce secreted IgA (sIgA) as IgA+ plasma cells. Reprinted from [54] with permission from Elsevier, Copyright 2017.

Figure 2 illustrates the possible events for mucosal immunization driven by an antigen/adjuvant nano-assembly as reprinted with permission from Bernocchi et al. 2017, reference [54]. The possible routes for an antigen/adjuvant nano-assembly are depicted from Figure 2A–E where there is direct capture by dendritic cell in Figure 2A, antigen diffusion through the cell junctions in Figure 2B, M cells performing the sampling of the antigen/carrier assemblies and directing them to appropriate cells in their M cell pocket in Figure 2C, epithelial cells performing endocytosis of the NP/antigen for further deliverance to local dendritic cells (DC) and T cells which are able to boost the immune response in Figure 2D. At last in Figure 2E, once in endosomes, NP could release antigens and be exocytosed as free, unloaded, NP (E1) and/or induce the endosomal escape of the antigens (E2) that would be processed as an endogenous antigen and presented by MHC-I (E3). In the other way, also on Figure 2E, NP could be degraded in endo/lysosomes (E4), and the released antigen be processed as exogenous and presented by MHC-II (E5). This could lead to DC and CD8+ T cell activation and/or priming. The activated DC from these pathways then migrate to germinal centers or directly to lymph nodes to activate CD4+ T cells that in turn activate B cells. They undergo an IgA+ phenotype switch, migrate by the blood flow to the effector sites and produce secreted IgA (sIgA) as IgA+ plasma cells.

Once NP/Ag undergo endocytosis by APC, the epitopes of processed antigens can be presented as complexes with either major histocompatibility complex I (MHC-I) or major histocompatibility complex II (MHC-II) [55,56]. In the case of cationic lipids, the versatile DODAB can be dispersed as nanosized, cationic bilayer fragments (BF) able to combine with oppositely charged antigens in general, driving the immune response to a cell-mediated one (Th1) [9,16]. In a certain sense, the DODAB BF are discoidal and open nanostructures instead of closed, vesicular bilayers or liposomes, and antigen is expected to become adsorbed from the electrostatic attraction all around the disk-like bilayer so that desorption and endosomal escape might allow MHC-I presentation after trafficking from the cytosol to the endoplasmic reticulum. This would agree with findings by Korsholm and coworkers [57]; they studied the mechanism of adjuvanticity for DODAB/OVA liposomes and found that these liposomes did not affect the maturation of murine bone-marrow-derived dendritic cells (BM-DCs) related to the surface expression of MHC-II, CD40, CD80, and CD86 but enhanced the uptake of OVA by BM-DCs via endocytosis; intraperitoneal injection of DODAB/OVA liposomes also enhanced the uptake of the antigen by peritoneal exudate cells and targeted the antigen preferentially to antigen-presenting cells, leading to enhanced uptake and presentation of antigen [57].

Nano-sized formulations enter the cells by endocytosis following the *endo*-lysosomal pathway before the protein is delivered and degraded in the endosomes; the resulting peptides are complexed with MHC-II and presented on cell surface for activation of CD4+ T helper cells, stimulating cytokine secretion and humoral antibody responses (Th2). When the nanostructure promotes the protein escape from the endosomes to the cytosol, the protein may be degraded in the proteasome with the peptidic products of the degradation carried by transporters of antigen processing to the endoplasmic reticulum where they combine with MHC-I. Cellular expression of peptide-associated MHC-I activates CD8+ T cells and cell-mediated immunity [58,59,60,61]. For effective control of tumors and pathogens by the immune system, neoplastic and infected cells must be targeted and destroyed by cytotoxic T lymphocytes (CTLs). While MHC-I conventionally present endogenous cytosolic antigens, the alternative pathway, termed cross-presentation, also allows the presentation of peptides derived from exogenous antigens by MHC-I [62]. As tumor antigens and pathogen-derived proteins are often not endogenously produced by antigen-presenting cells (APCs), this exogenous pathway is crucial for the generation of CD8+ CTL responses against these cell-associated antigens [63]. Enhancement of the targeting of exogenous antigens to the cross-presentation pathway may help develop effective vaccines against tumors, parasites, intracellular bacteria, and viruses. In summary, there are distinct intracellular routes for antigen uptake and presentation to attain CD4 and CD8 T cell activation and ideal antigen adjuvant systems should activate both of these pathways, thereby also inducing cross-presentation [58]. Since subunit vaccines are not effective in cytotoxic T cells activation, the association with adjuvants becomes crucial [64]. Interestingly, the antigen encapsulation in nanostructures (nanoparticles and bilayer nanodisks included) may direct the antigen presentation towards a different or combined immune response. This orientation can be affected by multiple factors, such as the mechanism of uptake, and is dependent upon the nanostructure physical properties such as the size, the outer surface charge, and also the inner particle charge. In our group, we observed that cationic nanodisks of DODAB BF complexed with the model antigen ovalbumin induced in vivo a large Th1 response and very low or absent humoral response [9,13,16], whereas NPs of PDDA/OVA, where the antigen was entangled with the cationic polymer PDDA, elicited potent Th2 humoral response in absence of the cell-mediated one [65]. Therefore, the ideal adjuvant should combine the ability of offering the antigen to be degraded inside the endosome with the ability to allow the antigen endosomal escape. Should we mix DODAB BF/antigen with PDDA/antigen to achieve the right balance between Th1 and Th2 responses?

**Figure 3:**
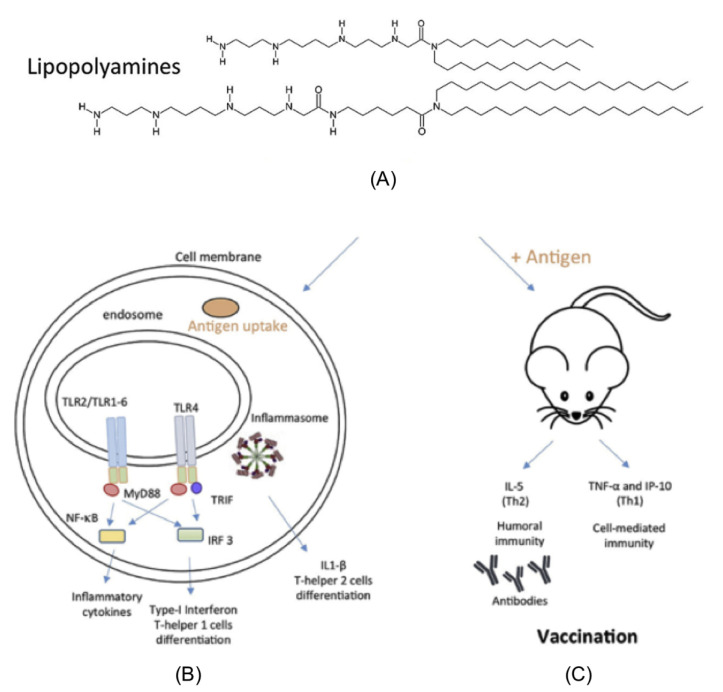
(**A**) Chemical structure of lipopolyamines (12 or 18 C). (**B**) Uptake of lipopolyamines alone or complexed with ovalbumin by cultured human cell lines transfected with Toll-like Receptors (TLRs), leading to 1) secretion of inflammatory and type-I interferon cytokines able to trigger a Th1 response (cell-mediated immunity); 2) secretion of the interleukin-1beta (IL-1β) able to induce a Th2 response (humoral immune response). (**c**) Uptake of lipopolyamines/antigen complexes in vivo by intraperitoneal macrophages induced secretion of interleukin-5 (IL-5) and humoral immunity plus tumor necrosis factor-alpha (TNF-α) and gamma-interferon inducible protein (IP-10) [71], typical inducers of Th1 response (cell-mediated immune response) by the cultured macrophages. Reprinted from [70] with permission from Elsevier, Copyright 2018.

The progress in gene or siRNA delivery to cells contributed substantially to the development of novel cationic lipids [66,67,68]. A particularly interesting class of cationic lipids is the lipopolyamines synthesized by Byk and coworkers in the 1990s aiming at DNA transfer to cells [69]. They were recently explored by Pizzuto and coworkers as single-component adjuvants able to elicit both Th1 and Th2 responses in absence of toxicity in vivo [70]. Figure 3 illustrates the ability of these polyamines to activate Toll-like receptor 2 (TLR2) and 4 (TLR4) besides inducing, in combination with OVA antigen, both IgG1 and IgG2a; OVA alone or Alum induced exclusively IgG1, and lipopolyamines induced both IgG1 and IgG2a antibodies production [70]. Figure 3A shows the chemical structure of the lipopolyamines. Figure 3B illustrates the uptake of lipopolyamines alone or complexed with ovalbumin by cultured human cell lines transfected with Toll-like Receptors (TLRs), leading to (1) secretion of inflammatory and type-I interferon cytokines able to trigger a Th1 response (cell-mediated immunity); (2) secretion of the interleukin-1beta (IL-1β) able to induce a Th2 response (humoral immune response). Figure 3C shows that the uptake of lipopolyamines/antigen complexes in vivo by intraperitoneal macrophages induced secretion of interleukin-5 (IL-5) and humoral immunity plus tumor necrosis factor-alpha (TNF-α) and gamma-interferon inducible protein (IP-10) [71], typical inducers of Th1 response (cell-mediated immune response) by the cultured macrophages.

On Figure 3 reproduced from Pizzuto and coworkers are shown two lipopolyamines with 12 or 18 carbon atoms in their alkyl chains; however, only the lipopolyamines with 12 or 14 carbon atoms in their alkyl chains activated TLR2- and TLR4-transfected cells, whereas the C18-lipopolyamine with very similar or identical polar head group activated only TLR2-transfected cells. The hypothesis cast to understand this was related to the fusogenic behavior of the lipopolyamines, since those with shorter lengths of the carbon chains (as those with C12 or C14) would be more fusogenic than those with long chains (as those with C18); thereby, the former would be taken up more easily by the cells. The possible toxicity in vivo of the lipopolyamine–OVA complexes was evaluated from determinations of liver enzymes alanine transaminase (ALT), aspartate transaminase (AST), and the inflammatory cytokine tumor necrosis factor-alpha (TNF-α) in the serum plus histological examination of liver slices of the injected mice post-injection; no toxicity was detected, neither in serum nor on liver slices. The TLR stimulation and secretion of pro-inflammatory and interleukin-1beta (IL-1β) cytokines suggested that the C12 or C14-polyamines would be promising one-component vaccine adjuvants eliciting both humoral and cell-mediated responses [70].

Aluminum adjuvants typically activate the inflammasome pathway and Th 2 response [72] so that alum combinations with TLR agonists are needed to induce the cell-mediated Th 1 response against pathogens [5]. Pizzuto et al. also demonstrated that lipopolyamines induced IP-10, IL-6, and IL-1β secretion in murine macrophages and TNF-α in murine and human macrophages. TNF-α and IL-6 are pro-inflammatory cytokines typical of the NF-κB induction. IP-10 is instead the signature of Type I IFN antiviral and T cell-stimulating response and is typical of the IRF induction. Finally, IL-1β secretion demonstrates the concomitant activation of the NF-κB pathway, which expresses pro-IL-1β, and of the inflammasome pathway that cleaves pro-IL-1 β. The activation of both TLR and inflammasome pathways combined with the carrier properties makes cationic lipid lipopolyamines excellent candidates as one-component vaccine adjuvants [70].

The mycobacterial cord factor trehalose-6,6′-dimycolate (TDM) present in the cell wall of mycobacteria and its synthetic adjuvant analog trehalose-6,6′-dibehenate (TDB) are glycolipids that trigger innate immunity. Bone-marrow-derived dendritic cells (BMDCs) stimulated with TDB induced Nlrp3 inflammasome-dependent IL-1β secretion; in vivo, in Nlrp3-deficient mice, recruitment of neutrophils by TDB was reduced, showing the essential role of the Nlrp3 inflammasome for the induction of an innate humoral immune response triggered by TDB [73].

In murine models of *Mycobacterium tuberculosis* (Mtb) infection, TDM administration drove the early pro-inflammatory M1-like macrophage response related to the granulomas of primary pathology; proinflammatory cytokines such as TNF-α, IL-1β, IL-6, and IL-12p40 were produced in lung tissue [74]. Furthermore, CD11b+CD45+ macrophages with a high surface expression of the pro-inflammatory CD38 and CD86 markers were found in lung lesions of mice at 7 days post-TDM introduction, but low phenotypic marker expression of anti-inflammatory M2-like markers CD206 and EGR-2 were present on macrophages. TDM played a role in establishment of the M1-like shift in the microenvironment during primary tuberculosis. Thus, the MTB cell wall cording factor TDM is a physiologically relevant and useful molecule for modeling early macrophage-mediated events during establishment of the tuberculosis-induced granuloma pathogenesis [74].

**Figure 4:**
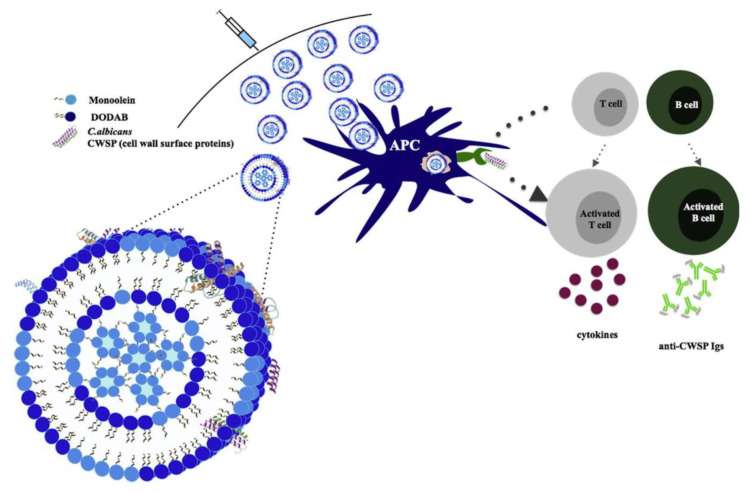
Activation of cell-mediated immunity and humoral response by DODAB/monoolein liposomes incorporating cell wall surface proteins of *Candida albicans* (CWSP). Reprinted from [77] with permission from Elsevier, Copyright 2015.

In order to improve fusion of cell membranes with DODAB bilayers, which are in the rigid gel state at room temperature [75], DODAB bilayer fluidity had to be increased by using DODAB combinations with other lipids and surfactants such as the glycolipid TDB [15,76], the surfactant monoolein (1-monooleoyl-rac-glycerol) [77], the C24:1 β-glucosylceramide [78] or the glycolipid de-O-acylated lipooligosaccharide (dLOS) as a booster vaccine against tuberculosis [79]. In particular, intranasal immunization with DODAB/TDB combined with influenza antigen A (H3N2) induced superior humoral and cell-mediated responses; there was an effective facilitation of uptake by DC, DC maturation in vitro, increased mucosal IgA production, increased IgG, IgG1, and IgG2b antibody titers in comparison with other formulations using cationic lipids after intranasal administration in vivo [80]. Immunization of mice with a mycobacterial fusion protein in DODAB-TDB liposomes induced a strong, specific Th1-type immune response characterized by substantial production of interferon-gamma mediated by CD4 T cells and high levels of IgG2b isotype antibodies [15]. The combinations of DODAB and monoolein improved the fusion of the liposomes with cell membranes, thereby allowing their use for mammalian cell transfection [81] and in vitro gene silencing [82]. These combinations also induced strong humoral and cell-mediated immune responses, producing antibodies (IgGs) against specific cell wall proteins of *Candida albicans* (CWSP) useful for fighting fungus infections [77,83]. Figure 4 illustrates the use and activity of DODAB/monoolein vesicles as adjuvants as reproduced from reference [77]. One should notice the inverted hexagonal phase of monoolein inside the liposome.

Another important line of research for vaccines against pathogens has been the use of cationic liposomes or DODAB bilayer fragments (BF) as adjuvants for intranasal immunization. The cationic DOTAP/DC-Chol liposomes combined with ovalbumin (OVA) were intranasally administered eliciting enhanced production of IgG antibodies in the serum (Th2 response) in immunized mice as well as mucosal IgA [84]. Immune responses for DODAB BF and alum complexes with outer membrane vesicles (OMV) of *Neisseria meningitidis* B administered by intranasal and subcutaneous routes in mice were compared; intranasal immunization produced a mixed Th1 and Th2 response, while subcutaneous immunization exhibited a Th1 profile only [27]. Non-replicating, nanometric membrane vesicles (MV) released both by Gram-positive and Gram-negative bacteria contain proteins, lipids, and nucleic acids that are effectively able to stimulate the innate and adaptive immune system [85,86]. In this regard, the cationic lipids can add extra adjuvanticity. Furthermore, multiple antigens can decorate these MV; for example, outer MVs from attenuated *S. typhimurium* was successfully decorated with one, two, or three antigens from *M. tuberculosis* (ESAT6, Ag85B, and Rv2660c) and major outer membrane protein epitopes from *Chlamydia trachomatis;* in vitro data showed that the antigen Ag85B delivered by outer MVs is able to be recognized and processed by dendritic cells and subsequently activate *M. tuberculosis*-specific T cells [87].

The development of effective intranasal vaccines is of great interest due to their potential to induce both mucosal and systemic immunity. Some oil-in-water nanoemulsion (NE) formulations containing various cationic and nonionic surfactants were used as adjuvants for the intranasal delivery of vaccine antigens. Association of NE droplets with the mucus protein mucin in vitro was important as were the cationic NE formulations that facilitated cellular uptake of the model antigen, ovalbumin (OVA), in a nasal epithelial cell line. NE-facilitated mucosal layer penetration and cellular uptake led to enhancement of the immune response [88].

**Figure 5:**
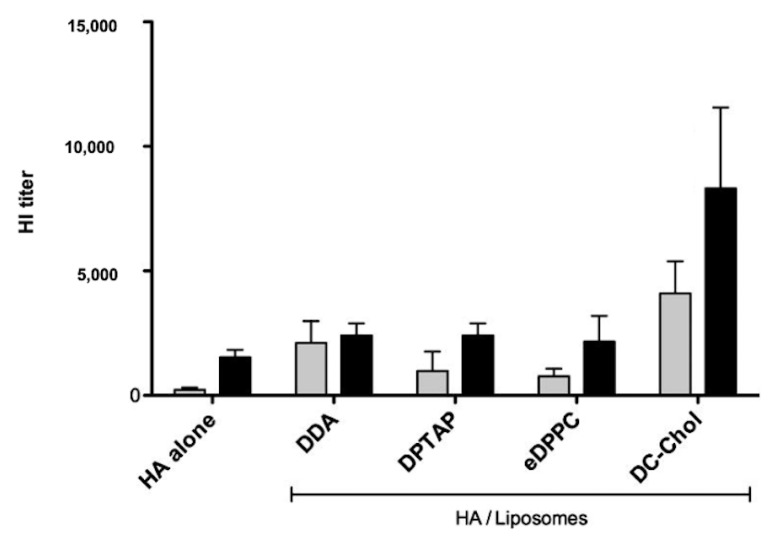
Hemagglutination inhibition (HI) assay for neutralizing antibodies against hemagglutinin (HA) elicited by combinations of HA with DDA/DPPC, DPTAP/DPPC, or DC-Chol/DPPC where DDA is dioctadecyldimethylammonium bromide, DPTAP is 1,2-dipalmitoyl-3-trimethylammonium-propane, DPPC is 1,2-dipalmitoyl-sn-glycero-3-phosphocholine, eDPPC is 1,2-diacyl-sn-glycero-3-ethylphosphocholine and DC-Chol is 3β-[*N*-(*N*′,*N*′-Dimethylaminoethane)-carbamoyl] cholesterol. Reprinted from [89] with permission from Elsevier, Copyright 2012.

In an interesting comparative study, several cationic lipids were evaluated regarding their effectiveness as humoral adjuvants while carrying the influenza antigen hemoagglutinin (HA) [89]. DDA or DODAB and other cationic lipids combined with a neutral lipid (DPPC) in a molar proportion of 1:1 were again evaluated as poor inducers of humoral response with exception of DC-Chol. The cationic liposomes contained a cationic compound (DDA or DODAB, 1,2-dipalmitoyl-3-trimethylammonium-propane DPTAP, DC-Chol, or 1,2-diacyl-sn-glycero-3-ethylphosphocholine (eDPPC) and a neutral phospholipid 1,2-dipalmitoyl-sn-glycero-3-phosphocholine (DPPC) and carried the influenza antigen HA; they were well characterized regarding hydrodynamic diameter, zeta potential, membrane fluidity, HA loading, and humoral immune response in subcutaneously immunized mice from the production of HA-specific antibodies by ELISA and HA-neutralizing antibodies by hemagglutination inhibition (HI) assay. Figure 5, reproduced from reference [89], shows that liposomes at 1:1 DC-Chol/DPPC combined with HA gave the inhibition of hemoagglutination titers that could be related to the highest IgG1 and IgG2a titers compared to the other liposomal HA formulations and HA alone. Moreover, increasing the proportion of cationic lipid increased the incorporation of HA and the immune response [89]. One should notice that the physical state of the cationic bilayers was the rigid gel state in all cases and the physical state of the DC-Chol/DPPC bilayers was not determined in reference [89].

The mRNA technology for vaccines [90] has been recognized as representing a transformative technology to control infectious diseases [91] and to fight cancer [92]. For example, while constructing an mRNA vaccine against influenza, the mRNA encoding the HA antigen of influenza A H1N1 virus was delivered by cationic lipid nanoparticles (LPN) and induced protective immune responses in mice. The lipid nanoparticles comprised several lipids such as DOTAP, 1,2-dioleoyl-sn-glycero-3-phosphoethanolamine (DOPE), and 1, 2-distearoyl-sn-glycero-3-phosphoethanolamine-N-(methoxy(polyethylene glycol)-2000) (DSPE-mPEG2000) (50:50:1 mol/mol) [93]. The system allowed also the covalent binding of mannose (Man) to the PEG moiety so that targeting of the mannose -cationic NPs (LNP-man) to the mannose receptors on antigen-presenting cells such as macrophages and dendritic cells improved the delivery efficiency of the assembly. These cationic lipid/mRNA NPs could protect their mRNA cargo from degradation by nucleases and deliver the m-RNA into cells by electrostatic adsorption and fusion with the cell membrane. LNP-Man contained DOTAP, DOPE, and DSPE-PEG-Mannose (50:50:1 mol/mol). This vaccine was properly tested from administration by the intra-nasal route and induced excellent protection against influenza. The important issue of lipid-based anticancer vaccines was recently reviewed [94].

In order to ascertain whether antigen depot or lymphatic targeting would benefit long-term immunological memory, OVA antigen was encapsulated with DOTAP cationic liposomes (LP) or DOTAP-PEG-mannose liposomes (LP-Man) to generate depot or lymphatic-targeted liposome vaccines, respectively [95]; in vivo imaging showed that LP accumulated near the injection site, whereas LP-Man accumulated in draining lymph nodes (LNs) and spleen enhancing the uptake by resident antigen-presenting cells. LP vaccines with depot effect induced higher anti-OVA IgG production than LP-Man vaccines on day 40 after priming but failed to mount an effective B-cell memory response upon OVA re-challenge after three months. In contrast, lymphatic-targeted LP-Man vaccines elicited sustained antibody production and robust recall responses three months after priming, suggesting that lymphatic targeting rather than antigen depot promoted the establishment of long-term memory responses [95].

**Figure 6:**
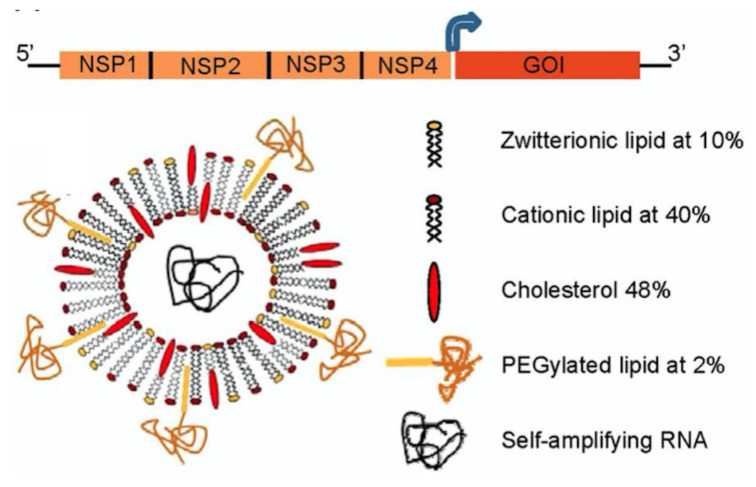
Self-amplifying RNA vaccines where this RNA derived from and alphavirus contains a 5′ cap, nonstructural genes (NSP1-4), 26S subgenomic promoter (dark blue arrow), the gene of interest, and a polyadenylated tail. The self-amplifying RNA was protected inside the cationic liposome with the composition shown in the legend on the right. Reprinted from [99].

Small interfering RNAs (siRNAs) are able to recognize a homologous mRNA sequence in the cell and induce its degradation; each siRNA molecule can inactivate several target RNAs in a sequence-specific manner [96]. The main problems in the development of siRNA-based drugs and vaccines for therapeutic use are the low efficiency of siRNA delivery to target cells and the degradation of siRNAs by nucleases in biological fluids [67]. Among the approaches used to deliver RNA are those based on non-saturated double-chained cationic lipids [97]. These lipids were shown to facilitate fusion with cell membranes [98]. Figure 6 shows the encapsulation of self-amplifying RNA based on alphavirus genome, which contains the genes encoding the alphavirus RNA replication machinery but lacks the genes encoding the viral structural proteins required for infection; the cationic liposome composition is also shown on the right as reproduced with permission from Geall and coworkers, 2012 [99]. The cationic lipid employed was 1,2-dilinoleyloxy-*N*,*N*-dimethyl-3-aminopropane (DLinDMA) with two double bonds per chain [98]. After immunization, replication and amplification of the RNA molecule occur exclusively in the cytoplasm of the transfected cells, thereby eliminating risks of genomic integration, cell transformation, and safety issues that occur for recombinant DNA, viral vectors, and pDNA vaccines. Furthermore, there is no need for crossing the nuclear membrane, a rate-limiting step for nonviral pDNA delivery.

Dendritic cells (DC) process and present antigens to T lymphocytes, inducing potent immune responses when encountered in association with activating signals, such as pathogen-associated molecular patterns. Monophosphoryl lipid A (MPL) is a ligand of the Toll-like receptor-4 and has been used in several studies on vaccines [100]. Using combined therapy against murine model tumors, both MPL and IL-12 were included in cationic DOTAP liposomes for intratumoral injection [101]. In 4T1 murine model of breast cancer, the injection decreased cellular proliferation and increased serum levels of IL-1β and TNF-α. The addition of recombinant IL-12 further suppressed tumor growth and increased expression of IL-1β, TNF-α, and interferon-γ. IL-12 also increased the percentage of cytolytic T cells, DC, and F4/80(+) macrophages in the tumor. The combination of MPL and IL-12 elevated the levels of nitric oxide synthase 7-fold above basal levels in the tumor and caused cell cycle arrest and apoptosis, also inhibiting the growth of untreated tumor in the same animal and revealing the systemic activity of the formulation [101].

**Figure 7:**
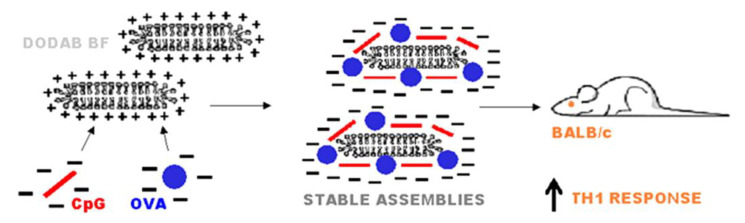
Schematic representation of cross sections for DODAB bilayer fragments (DODAB BF) used for carrying ovalbumin (OVA) and CpG agonist of Toll-like receptor 9. The final assemblies were anionic and directed excellent Th1 response in mice immunized subcutaneously. Curiously, addition of CpG to the assembly did not improve the immune response; DODAB was effective by itself. In addition, the nanosize of the assemblies was more important than the charge. Reprinted from [16] with permission from Elsevier, Copyright 2012.

In another very interesting approach, sterically stabilized nanodisks based on high-density lipoproteins (HDL) carried MPL, CpG (ligand of Toll-like receptor-9), and antigen for personalized cancer immunotherapy; synthetic high-density lipoprotein (sHDL) nanodisks were composed of phospholipids and apolipoprotein A1 (ApoA1)-mimetic peptides (the peptides were named 22A because they were synthesized as 22-mer peptides, derived from the repeat α-helix domain of ApoA1) [102]. Thereby the endogenous role of HDL as a nanocarrier for cholesterol was explored in synthetic HDL that carried cholesteryl-CpG, neo-antigens, and tumor Ag peptides (neo-antigens identified via tumor DNA sequencing) to produce homogeneous, stable, and ultrasmall nanodisks in less than two hours at room temperature; nanodisks promoted co-delivery of Ag/CpG to draining lymph nodes; prolonged Ag presentation on antigen-presenting cells (APCs); elicited striking levels of broad-spectrum antitumor T-cell responses; and significantly inhibited tumor growth, also eradicating established tumors [102]. Cationic nanodisks of DODAB, also called DODAB bilayer fragments (BF), have also been used as adjuvants for carrying several antigens, CpG agonist, and oligonucleotides [16] directing excellent Th1 response and also Th2, depending on the administration route [see references [21,25,28]. Figure 7 schematically represents cross-sections of DODAB nanodisks carrying CpG and ovalbumin (OVA). DODAB BF have two major strategic advantages when compared to more sophisticated formulations: (1) DODAB is possibly the less expensive synthetic cationic lipid available nowadays, (2) DODAB dispersion as open bilayers in water solution can be rapidly performed by sonication with a macrotip, and (3) the nanometric size of the DODAB bilayer disks allows direct stimulation of APC in the draining lymph nodes [103].

Whereas DODAB BF harboring CpG did not improve the adjuvanticity of DODAB BF in vivo [16], DOTAP/DC-chol liposomes harboring CpG ODN as a mucosal adjuvant induced both antigen-specific mucosal IgA responses and balanced Th1/Th2 responses so that the combination resolved adverse effects of CpG ODN as mucosal adjuvant by means of dose minimization [104].

Human papillomavirus (HPV) is the most common sexually transmitted biological agent and causes precancer lesions and cancer; three prophylactic HPV vaccines targeting high-risk HPV types are available in many countries worldwide: 2-, 4- and 9-valent vaccines; all three of the vaccines use recombinant DNA technology and are prepared from the purified L1 protein that self-assembles to form HPV type-specific empty shells [105]. There are a few instances of using cationic lipids to formulate vaccines against HPV. DOTAP/oncoprotein E7 of papillomavirus was evaluated for its anti-cancer activity; E7 peptide formulated with DOTAP induced migration of activated dendritic cells (DC) to the draining lymph node (DLN) and efficiently generated functional antigen-specific CD8+ T lymphocyte infiltration and apoptosis at tumor sites; the effect did not change by adding CpG to the same formulation [106]. Efficient eradication of tumors in mice was also achieved using combinations of DOTAP/DOPE cationic liposomes with synthetic long peptides (SLP) derived from OVA alone or combined with different Toll-like receptors ligands including CpG; a single intradermal tailbase vaccination of tumor-bearing mice with a low dose of E7/poly(I:C)-liposomes led to complete clearance of the tumors in 100% of the mice; therapeutic vaccination with SLP could be clinically effective against HPV-induced premalignant lesions; induced antigen-specific CD8+ and CD4+ T cells and in vivo cytotoxicity against target cells after intradermal vaccination; at a low dose (1 nmol) of SLP, our liposomal formulations significantly controlled tumor outgrowth in two independent models (melanoma and HPV-induced tumors) and even cured 75%–100% of mice of their large established tumors; cured mice were fully protected from a second challenge with an otherwise lethal dose of tumor cells, indicating the potential of liposomal SLP in the formulation of powerful vaccines for cancer immunotherapy [107].

SLP-loaded (1,2-dioleoyl-3-(trimethyammonium) propane)-based cationic formulation as a therapeutic cancer vaccine was tested against two independent tumor models. The OVA-derived SLPs containing CTL and Th epitopes were loaded into DOTAP- based cationic liposomes combined with different TLR ligands [poly(I:C), Pam3CysK4, CpG], and the most potent formulations were applied in a foreign antigen (OVA)-expressing melanoma model. In an independent setting, HPV16 E7 SLP was formulated in the same liposomal system and analyzed as a therapeutic vaccine in the TC-1 HPV+ tumor model; both formulations were highly effective in the induction of cellular immunity and tumor control [107].

The humoral and cellular immune responses induced in mice against hepatitis B virus surface antigen (HBsAg) were examined when the antigen was either adsorbed to aluminum hydroxide or administered with DC-Chol. DC-Chol induced cellular immune responses to HbsAg and a balanced Th1/Th2 response, which enabled mice to overcome the inherited unresponsiveness to HBsAg encountered with aluminum-adjuvanted vaccine. Thus, the DC-Chol provided a signal to switch on both Th1 and Th2 responses for vaccination against hepatitis B virus [108].

An early model study on trafficking of cationic-liposome-DNA complexes in the cells attempted to reveal by electron microscopy the intracellular fate of gold-labeled structures. Cells treated with DOTMA liposome-DNA complexes demonstrated endocytosis of the liposome–DNA complexes in coated pits, which were seen in early endosomes, late endosomes, and lysosomes. In isolated alveolar type II cells, the gold-labeled DOTMA lipid apparently mixed with the contents of lamellar bodies. In most cells, gold particles were dispersed throughout the cytoplasmic matrix. In a small proportion of cells, a membrane system resembling the endoplasmic reticulum developed within the nucleus; this novel structure was also present in isolated nuclei from cells and then mixed with DOTMA-containing liposomes [109].

DNA vaccination technologies have been important in several areas despite the difficulties involving DNA transfection efficiency, prevention of DNA degradation, APC targeting, and enhancing DNA escape from endo/lysosomal compartments and attachment of virus-derived nuclear localization sequences facilitating nuclear entry of the DNA [110]. For example, DNA vaccines provide an attractive technology platform against anthrax bioterrorism agents; monovalent and bivalent anthrax plasmid DNA (pDNA) vaccines encoding genetically detoxified protective antigen (PA) and lethal factor (LF) proteins were formulated in cationic lipids, and immune responses after two or three injections of cationic lipid-formulated PA, PA plus LF, or LF pDNAs were at least equivalent to two doses of anthrax vaccine adsorbed (AVA). High titers of anti-PA, anti-LF, and neutralizing antibody to lethal toxin (Letx) were achieved in all rabbits. All animals receiving PA or PA plus LF pDNA vaccines were protected. In addition, 5 of 9 animals receiving LF pDNA survived, and the time to death was significantly delayed in the others. Groups receiving three immunizations with PA or PA plus LF pDNA showed no increase in anti-PA, anti-LF, or Letx neutralizing antibody titers postchallenge, suggesting little or no spore germination. In contrast, titer increases were seen in AVA animals and in surviving animals vaccinated with LF pDNA alone. Preclinical evaluation of this cationic lipid-formulated bivalent PA and LF vaccine is complete, and the vaccine has received U.S. Food and Drug Administration Investigational New Drug allowance [111].

Early work involving cationic liposomes to carry plasmid encoding antigens revealed that the liposomes indeed protected the liposome-entrapped DNA from degradation in vivo, thereby resulting in greater antibody responses against the encoded antigen when compared with naked DNA, both given via the subcutaneous route; T-cell responses from analysis of interferon-γ and interleukin-4 levels in the spleens of mice treated with liposomes/DNA were also significantly higher than those measured in mice treated similarly with naked DNA [112,113,114]. More recently, Perrie and coworkers gave an excellent summary of the possible fate of cationic liposomes/DNA (L/DNA) assemblies injected by the subcutaneous route [115]. L/DNA assemblies injected locally are taken up by APC penetrating the site of injection or in the lymph nodes draining the injected site; the clearance of L/DNA from the site of injection depends on L/DNA size; the smaller the size, the more rapid the clearance through the anatomical barriers [116]. The L/DNA become dispersed throughout the lymph node either permeating along the sinuses or being taken up by cells such as macrophages; once within cells, L/DNA are digested by the lysosomes, leaving their contents within the lysosomes or escaping this degradation via fusion with the endosomal membrane (which happens due to the fusogenic lipid DOPE present in the liposomal bilayer) [117,118,119]. Thereby DNA is displaced from the complex and released into the cytosol for eventual episomal transfection so that cationic lipid and DOPE favor liposome-mediated -DNA immunization [115,117,118]. The possible fates of nanosized adjuvant/antigen assemblies were thoroughly discussed by Smith and coworkers, 2013 [120].

Lecithins are components of cell membranes consisting of combinations of phosphatidylcholine (PC), phosphatidylethanolamine (PE), phosphatidylserine (PS), and phosphatidylinositol (PI), plus other substances such as triglycerides and fatty acids; in particular, soy lecithin contains 21% PC, 22% PE, and 19% PI, along with other components. Lecithins are widely used for dispersing, emulsifying, and stabilizing a variety of pharmaceuticals often included in intramuscular and intravenous injectables, parenteral nutrition formulations, and topical products [121]. Hexadecyltrimethyl ammonium bromide (CTAB) surfactant added to lecithin nanoparticles yielded cationic particles with diameters in the range of 100–200 nm where soy lecithin was the matrix material and CTAB, the outer surfactant. The zeta potential of the particles was positive, reached a value of about 40 mV at a CTAB concentration of 2.5 mM, and was used to adsorb plasmid DNA and transfect cells efficiently, representing a potential carrier for DNA vaccines [122].

Cationic liposomes are commonly used as a transfection reagent for DNA, RNA, or proteins and as a co-adjuvant of antigens for vaccination trials. A high density of positive charges close to cell surface is likely to be recognized as a signal of danger by cells or contribute to trigger cascades that are classically activated by endogenous cationic compounds, though carrier/protein or carrier/nucleic acid might have anionic charges and still trigger significant immune responses. There are several cellular pathways, like pro-apoptotic and pro-inflammatory cascades, that can be induced by cationic liposomes, depending on their nature, size, and structural properties (nature of the lipid hydrophilic moieties, hydrocarbon tail, mode of organization) [123]. Their use and design for specific applications such as gene transport or as adjuvants certainly require more knowledge on their structure–function relationship. Excellent reviews are available on this nano-era regarding applications of nanotechnology in immunology [120,124], progress in prophylactic and therapeutic nanovaccines [124], cancer nano-immunotherapy [125], nanomaterial interactions with the immune system [126], and liposomes formulations for vaccines [127,128].

## 3. Assemblies Based on Cationic Polymers

Similarly to cationic lipids, cationic polymers constitute another important class of cationic adjuvants despite their often-reported dose-dependent cytotoxicity that requires dose minimization [9,10,65,129,130,131,132,133]. They easily combine with oppositely charged proteins [40,134]. Biodegradable polymeric particles of poly (lactic-co-glycolic acid) (PLGA) have been combined with cationic surfactants or lipids or polymers (CTAB, DODAB, polyethyleneimine (PEI) or ε-poly-l-lysine (PLL)) for improving antigen adsorption, colloidal stability, and the immune response [135,136]. Nanocomplexes of PEI and antigens (influenza hemagglutinin or herpes simplex virus type-2 glycoprotein D) delivered by the mucosal route activated APC in vivo, promoting dendritic cell trafficking to draining lymph nodes besides eliciting a potent immune response against the viral subunit glycoproteins; a single intranasal administration elicited robust antibody-mediated protection [137]. Systemic administration of the same antigens with PEI induced both Th1/Th2 immune responses and higher titers of both antigen-binding and -neutralizing antibodies than alum [138].

The cationic antimicrobial polymer poly (diallyldimethylammonium chloride) (PDDA) is a poly-cation [139,140,141] able to combine with bovine serum albumin (BSA), yielding NPs with diameters around 50 nm [142,143]. In combination with HIV-1 DNA, nanorods of gold yielded particles of gold/PDDA/DNA, which elicited a Th2 response that was higher than the one obtained using PEI or cetyltrimethylammonium bromide (CTAB). Stimulated cellular and humoral immunity, as well as T cell proliferation, was obtained in comparison with naked HIV-1 Env plasmid DNA treatment in vivo [144]. Recently, NPs of PDDA/ovalbumin were prepared, characterized by their physical properties, and evaluated as stimulators of the OVA-specific immune response [65]. Dynamic light scattering (DLS) showed that these cationic PDDA/OVA NPs at reduced doses of cationic polymer had low size, positive zeta-potential, low polydispersity, good colloid stability, and low cytotoxicity against mammalian cells in culture eliciting potent Th2 OVA-specific immune response (high OVA-specific IgG1 and low OVA-specific IgG2a production); the OVA-specific antibody production was even higher than the one elicited by Al(OH)_3_/OVA [65].

**Figure 8:**
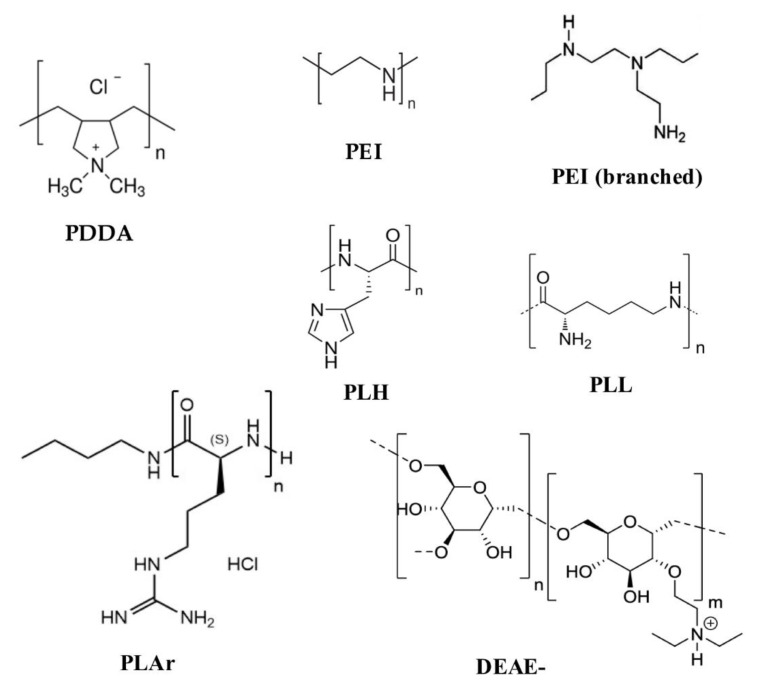
Chemical structures of some cationic polymers: poly (diallyldimethylammonium chloride) (PDDA), linear polyethyleneimine (PEI), branched polyethyleneimine (PEI), poly-l-arginine (PLAr), poly-l-lysine (PLL), poly-l-histidine (PLH), diethylaminoethyl-dextran (DEAE-).

Polycations combine spontaneously with molecules of opposite charge like proteins and nucleic acids [65,137,142]; thus, the main use of polycations in the biomedical field is the delivery of bioactive molecules including DNA, RNA, and protein [145]. Polycations as adjuvants can be used in a variety of assemblies, ranging from the simple complexation of polymer with antigen driven by electrostatic interactions [65,138,146] to the use of the polymers as particle coatings or particle cores [147]. The spontaneous complexation of polycations with negatively charged antigens is the simplest way to use these systems as antigen carriers. Figure 8 shows the chemical structures of some cationic polymers that have been used as antigen carriers.

Polyethyleneimine (PEI) is an organic, hydrophilic, and cationic polymer that displays a strong positive charge density promoting the combination with negatively charged molecules such as DNA, negatively charged antigens, or plasma membranes [146]. An interesting quality of PEI is its ability to leak from endosomes after cell internalization due to its capacity for avoiding the endosomal acidification; the high number of nitrogen atoms in the PEI molecule makes the polymer an excellent buffer also at low pH [148]. This proton sponge effect is due to deprotonated PEI amino groups binding protons as they are pumped into the lysosome, resulting in the influx of Cl^–^ ions and water with lysosome osmotic swelling and stretching of the PEI molecule itself due to repulsion between protonated amino groups; thereby, there is rupture of the lysosomal membrane with release of lysosomal contents into the cytoplasm, the so-called endosomal escape leading to a cellular, Th1 response [149,150]. PEI as an adjuvant protects antigens from enzymatic degradation [151], activates the inflammasomes, up-regulates the transcription factor called interferon regulatory factor 3 (Irf-3) [137] and also other immunostimulatory genes [152], induces Th1 immune response associated with endosomal escape and cross-presentation [153]; administered by the mucosal route, it improves the uptake of antigen and the activation of APCs [137,154]. PEI promotes dsDNA release by host cells triggering the Irf-3-dependent signaling [137]; Irf-3 is a transcription factor related with the activation of innate response by means of the synthesis of type I interferon [155]. Besides Irf-3, PEI activates Nlrp3 inflammasomes, a critical component of the innate immune system, which directs the immune response toward a Th2-biased type [137,138]. PEI as an adjuvant can induce Th1-, Th2-, or Th1/Th2- biased immune response; although PEI has the proton sponge effect to carry out the lysosomal escape, sometimes the escape does not occur, and antigens are presented via MHC class II, resulting in a Th1/Th2 or Th2 response [137,138,146]. PEI/antigen administered by intranasal route induced Th2 response [137], while PEI/antigen administered by a subcutaneous route yielded a Th1/Th2 mixed response [138].

Aiming at improving the mucosal response against major viral pathogens, glycoproteins derived from influenza A virus, herpes simplex virus type-2, or HIV-1 combined with PEI as particles in dispersion, administered as a single intranasal dose induced a robust protection from further lethal viral infection, which was superior to the one elicited by cholera toxin as an experimental mucosal adjuvant; these PEI/antigen nanoparticles were efficiently taken up by APCs in vitro, while in vivo they enhanced the DCs trafficking to draining lymph nodes. The nasal immunization with the recombinant envelope glycoprotein gp140 from HIV-1 carried by PEI induced high titer of antigen-specific IgA in vaginal lavages, demonstrating that nasal immunization can induce a systemic immune response. In Nlrp3-knockout mice, PEI/gp140 complexes elicited a Th1-biased immune response, suggesting that in normal mice the NPs activated the inflammasome Nlrp3 towards a Th2-type response [137]. In continuation, four different PEI polymers such as linear PEI (40 and 160 kDa) and branched PEI (25 and 750 kDa) were combined with the gp 140 from HIV-1; after immunization, all elicited similar responses characterized by a moderate Th1-biased response. The comparison of PEI with alum showed significantly improved performance of PEI compared to alum. PEI-induced immune response was characterized by an intermediate IgG1/IgG2a endpoint titer ratio, indicating a mixed Th1/Th2 immune response as corroborated from analysis of cytokines profile using antigen-re-stimulated splenocytes from mice immunized with gp140 glycoprotein and PEI. Significant amounts of Th1 cytokines IL-2, TNF-α, and GM-CSF and the Th2-associated cytokine IL-5 were determined [138].

Eliciting a cell-mediated immune response is necessary for achieving effective vaccines against cancer and other major diseases like malaria; thus adjuvants able to enhance the antigen presentation via MHC I are needed. Chen and coworkers reported that PEI/OVA NPs, obtained from mixtures of PEI and OVA, promoted cross-presentation through the major MHC class I pathway. The mouse bone-marrow-derived dendritic cells stimulated in vitro with PEI/OVA NPs resulted in improved and more frequent OVA(257-264)/MHC I complex presentation on dendritic cell surfaces. Besides, DCs pulsed with PEI/OVA NPs but not those pulsed with OVA alone showed significant capacity to stimulate T cells [153]. Using DNA as antigens is an encouraging alternative for designing anticancer therapeutic vaccines. Complexes formed by OVA plasmid and PEI (PEI/DNA) were administered to animals, and the corresponding immune response and antitumor activity were assessed. Animals injected with the PEI/DNA complexes displayed antigen-specific cell lysis, and there was increased antigen presentation via MHC class I and significant lymphocyte infiltration at the intra-tumor inoculation sites; importantly, the vaccine injected either before or after the tumor cell inoculation repressed the tumor growth and increased the survival rate of animals [156].

**Figure 9:**
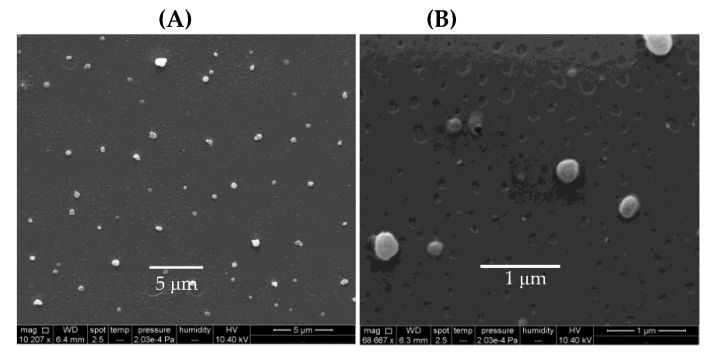
Scanning electron micrographs of PDDA/OVA NPs assembled at 0.1 mg·mL^−1^ OVA and 0.01 mg·mL^−1^ PDDA obtained under low (**A**) and high magnification (**B**). At the low PDDA dose employed, PDDA cytotoxicity was not significant against cells in culture. Reprinted from [65].

Poly (diallyldimethylammonium chloride) (PDDA) is a synthetic and linear polycation that combines well with DNA [157,158] or protein [142,143]. Figure 9A,B show scanning electron micrographs of PDDA/OVA nanoparticles (NPs) under low and high magnification, respectively; these NPs elicited OVA-specific Th2 response in immunized mice, which was superior to the one elicited by alum [65].

Biomedical uses for PDDA have mostly focused on the design of biosensors [159], transfection [158], or antimicrobial chemotherapy [139,140,141,160,161,162,163] probably due to its high toxicity [65,129]. PDDA, when combined with OVA, in water, formed cationic, nano-sized, and stable NPs that displayed a dose-dependent cytotoxicity, which could be easily controlled from the use of low doses. Interestingly, PDDA/OVA NPs induced a potent Th-2-type immunity (high ratio IgG1:IgG2a) and elicited an OVA-specific IgG1 antibody production higher than the one induced by OVA or Al(OH)_3_/OVA; PDDA/OVA NPs displayed low cellular immune response as determined from footpad swelling tests for detecting the delayed-type hypersensitivity reaction (DTH) and the low elicited production of IgG2a quantified in serum, both in immunized mice [65].

**Figure 10:**
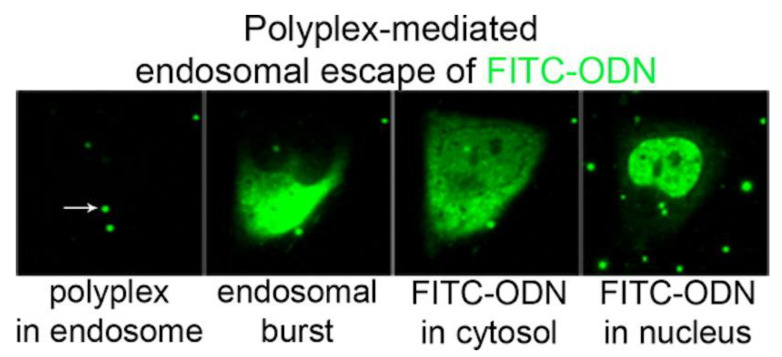
PEI-mediated cytosolic delivery of oligonucleotides occurs by endosomal bursting. HeLa cells were incubated with polyplexes carrying fluorescein isothiocyanate labeled oligo-deoxy nucleotides (FITC-ODNs) in green and monitored by time-lapse microscopy. Following internalization of the poly (ethylene imine) (PEI) polyplexes in endosomes, endosomal bursting occurs causing the release of ODNs into the cytosol. After an initial appearance throughout the cytosol, the ODNs rapidly accumulate in the nucleus. Reprinted from [165] with permission from 2013 American Chemical Society.

An important biomolecule that can be carried by cationic polymers is DNA, essential for DNA vaccines despite its relative delivery inefficiency when compared to viral vectors [164]. Cell internalization of the polyplexes (cationic polymer/DNA) and subsequent release of their cargo requires translocation across endosomal and/or nuclear membranes, a determinant factor for therapeutic efficiency, and hence, potential clinical impact. Polyplexes or lipoplexes (cationic lipid/DNA) essentially follow a similar intracellular route once captured by endocytosis [165,166]. Figure 10 shows the intracellular trafficking of a fluorescently labeled oligo-deoxynucleotide (ODN) carried in PEI/fluorescein isothiocyanate-labelled ODN (FITC-ODN) polyplexes; fluorescence is firstly inside the endosome, then in the cytosol after escaping from the endosome due to the endosomal burst, and finally in the cell nucleus as reproduced from ur Rehaman et al. 2013 [166]. The limited efficiency of ODN delivery to the nucleus relates to the fact that most endosomes did not burst.

Diethylaminoethyl-dextran (DEAE-) polymer is a quaternary ammonium compound that contains three basic groups with different pKa values [167]. The polymer facilitates the adsorption and penetration of viral particles or bacteria into cells, suggesting that it is adequate for delivering antigens into the APCs [168]. DEAE- has delivered Venezuelan equine encephalomyelitis virus [169] and whole-cells *Vibrio cholerae* Inaba and Ogawa serotypes vaccines [170], although its adjuvant properties have been explored mainly for use in vaccines for veterinarian treatment [171]. DEAE-Dextran mixed with formalin-inactivated Venezuelan equine encephalomyelitis virus exhibited a significant adjuvant effect on the primary immune response in rhesus monkeys [169]. In a whole-cell vaccine, DEAE- combined with *Vibrio cholerae* produced a higher and longer-lasting antibody titer than the one elicited by vaccines without adjuvant; furthermore, there was a greater protection against cholera re-infection [170]. In a breast cancer model, DEAE- induced the production of IFN-beta inhibiting the gene expression of the vascular endothelial growth factor (VEGF) gene and the NOTCH1 gene both related to angiogenesis and tumorigenesis [172].

Poly (2-aminoethyl methacrylate) (PAEM) homopolymers with defined chain length and narrow molecular weight distribution were synthesized using atom transfer radical polymerization (ATRP) so that PAEM of different chain lengths (45, 75, and 150 repeating units) showed varying strength in condensing plasmid DNA into narrowly dispersed nanoparticles. Longer polymer chain length resulted in higher levels of overall cellular uptake and nuclear uptake of plasmid DNA, but shorter polymer chains favored intracellular and intranuclear release of free plasmid from the polyplexes. Using a model antigen-encoding ovalbumin plasmid, transfected DCs activated naïve CD8(+) T cells to produce high levels of interferon-γ. Efficiency of transfection, DC maturation, and CD8(+) T cell activation showed varying degrees of polymer chain-length dependence, showing the importance of using structurally defined cationic polymers as carriers for DNA vaccines [173]. This model study emphasized the importance of well-defined chain length for cationic polymers in DNA vaccines; this type of cationic polymer poly (2-aminoethyl methacrylate) was also recently reviewed for exploring structure–function relationship while delivering DNA [174]. Excellent reviews are available on micro- and nanoparticles for DNA vaccine delivery [175] and on molecular delivery of plasmids for genetic vaccination [176].

Polyaminoacids are another important class of polycations used for carrying antigens in vaccine formulations [177]. Like other polycations, one major advantage of using them is their ability to combine spontaneously with molecules of negative charge, a phenomenon driven for electrostatic interactions. Within the group of polyaminoacids, poly-l-lysine and poly-l-arginine are among the most studied as adjuvants. For instance, poly-l-arginine on the surface of microcapsules obtained by layer-by-layer or spray-drying techniques supports the particles’ uptake by the APCs [178,179]. Promising research reported the use of poly-l-arginine for carrying tumor antigen-derived peptides for synthesizing a synthetic antitumor vaccine; the work described that the subcutaneous injection of a mixture of poly-l-arginine and peptides induced a large number of antigen-specific T cells detectable in the systemic circulation for more than four months. Another important finding reported by the authors was the presence of numerous APCs loaded with antigen in the draining lymph nodes of vaccinated animals, suggesting that poly-l-arginine is a suitable carrier for targeting antigens into the lymph nodes. An additional relevant result is the absence of antibodies against poly-l-arginine, allowing this compound to be used for numerous booster injections [180].

Employing TLR agonists as adjuvants is an attractive alternative for stimulating a specific type of immunity like a Th1-biased response. Negatively charged TLR agonists could also be easily attached to polycations by electrostatic interactions [138]. The adjuvant activity of combinations between CpG ODN (ligand for Toll-like receptor 9) and different polycations such as poly-l-arginine, poly-l-lysine, poly-l-histidine, or chitosan in an OVA vaccination model, as well as poly-l-arginine and poly-l-histidine, but not poly-l-lysine or chitosan, improved efficiently both the IgG antibody production and the number of splenocytes secreting IFN-gamma after T CD8+ ex vivo stimulation. CpG-ODN/poly-l-arginine is better than complete Freund’s adjuvant and aluminum salt as adjuvants and did not induce local toxicity [181]. The assembly of poly-l-arginine, CpG-ODN, and synthetic peptides induced an improved peptide-specific immune response as compared to the application of peptides with either of the immunomodulators alone. Poly-l-arginine synergized with oligodeoxynucleotides containing CpG-motifs (CpG-ODN) for enhanced and prolonged immune responses and prevented the CpG-ODN-induced systemic release of pro-inflammatory cytokines; CpG-ODN are known to be potent inducers of predominantly type 1-like immune responses, while polycationic amino acids, facilitate the uptake of antigens into antigen-presenting cells (APCs). The potentially harmful systemic release of pro-inflammatory cytokines induced upon injection of CpG-ODN was inhibited and long-lasting, and high numbers of antigen-specific T cells could be observed from fluorescence-labeled OVA even after only one injection of the vaccine [182].

Cationic polymers are also used for coating or functionalizing different types of NPs, namely, magnetic NPs, metallic NPs, ceramic NPs, hydrophobic NPs, or biocompatible polymer-based NPs. For instance, PEI has been used for coating SPIONs [19,183,184], silica NPs [185], or gold NPs [144,186]. Cationic and nanosized PEI-coated SPIONs in vitro activate macrophages and polarize them to an M1-like phenotype [184]. PEI-coated SPIONs obtained from the sonication of iron oxide suspension and PEI solution was used for carrying the plasmid-malaria DNA vaccine encoding VR1020-PyMSP119; the intraperitoneal administration of the complex displayed high titers of antigen-specific IgG1 and IgG2a and improved the immunological memory after vaccination [19]. Another example of metallic nanoparticles functionalized with polycations was reported for Xu and coworkers; they coated gold nanorods with the polycations PEI or PDDA or with the surfactant CTAB and attached therapeutic HIV-1 DNA-vaccine. Gold nanorods coated with both polycations show the best capacity for packing DNA, and they were uptaken by DCs in a higher level than the ones coated with CTAB; polycation-coated gold nanorods induced a significant increase of the cellular and humoral immunity as determined by antibody titers (IgG1/IgG2 ratio) and T cell proliferation. It was also described that PDDA enhances a Th2 whereas PEI induces a Th1/Th2 immune response [144].

In situ silicifications and capping treatment produced PEI–silica–PEI coatings on Japanese encephalitis virus vaccine maintaining its immunogenicity better than the vaccines without adjuvant; this fact could be relevant in cases where keeping the vaccine under refrigerated condition is difficult [187]. PEI coating mesoporous silica micro-rods (MSR-PEI) significantly enhanced the antigenicity of a peptide antitumor vaccine. The antigen was coupled to the microstructure by simple adsorption enhancing host DCs activation and T cell response compared to controls; MSR-PEI/E7-peptide eradicated large established TC-1 lung tumors in ~80% of mice and generated immunological memory; MSR-PEI vaccine, using a pool of neoantigens, eliminated established lung metastases and controlled tumor growth [188].

Carboxylated polystyrene particles conjugated covalently with poly-l-lysine carried a DNA vaccine improving the immune Th-1 response; C57BL/6 mice were immunized with the NPs carrying an OVA plasmid yielding a high level of activated CD8 T cells and OVA-specific antibodies. The animals immunized with the poly-l-lysine-coated polystyrene NPs generated a significant antitumor response evidenced by the inhibition of tumor growth after challenging with the OVA expressing EG7 tumor cell line; NPs with diameters of 50 nm yielded the best activity [189].

In summary, polycations can be excellent adjuvants for vaccines, but a quantitative determination of toxicity should always be performed for determining the minimal doses where toxicity is absent and the adjuvant effect still occur; this includes also the equally toxic cationic polyaminoacids. One should notice also that cationic polymers in general are potent antimicrobials as often reviewed [139].

## 4. Hybrid Cationic Assemblies of Biocompatible Polymer/Cationic Polymer

Hybrid cationic polymer/biocompatible polymer assemblies are useful for improving the characteristics of delivery systems. For example, the use of biocompatible polymers decreases the toxicity of the systems, whereas the cationic polymers improve the colloidal stability, antigen loading capacity, and APCs internalization. Some researchers have used this approach for achieving improved delivery systems [136,190,191].

Biocompatible synthetic or natural polymers can improve body functions without interfering with its normal functioning or triggering side effects [192,193,194]. Some examples of biocompatible polymers are poly (lactic-co-glycolic acid) [195], poly (ε-caprolactone) (PCL) [196], poly (lactic acid) [197], poly (3- hydroxybutyrate-co-3-hydroxyvalerate) (PHBV) [198], chitosan [199,200], cellulose [201] and poly (acrylates) including poly (methyl methacrylate) (PMMA) [202,203]. Neutral or anionic biocompatible synthetic or natural polymers as polylactide-co-glycolide microparticles have been cationized with cationic lipid DODAB or cationic surfactant CTAB to assure combination with the oppositely charged DNA antigens to design DNA vaccines [135,204]. In another instance, DODAB cationic bilayer surrounded polystyrene sulfate particles available over a range of diameters [205,206,207] or silica particles [208,209]; recently optimal coverage with single cationic bilayers on the anionic polymeric or inorganic particles was achieved [207,209]. Synthetic biocompatible polymers such as PMMA were also hybridized with cationic polymers or cationic lipids or surfactants [160,161,162,163,210,211,212,213]. In summary, a variety of model hybrid and cationic delivery systems are already available to combine with a myriad of oppositely charged antigens for vaccine design [25,163,214,215].

**Figure 11:**
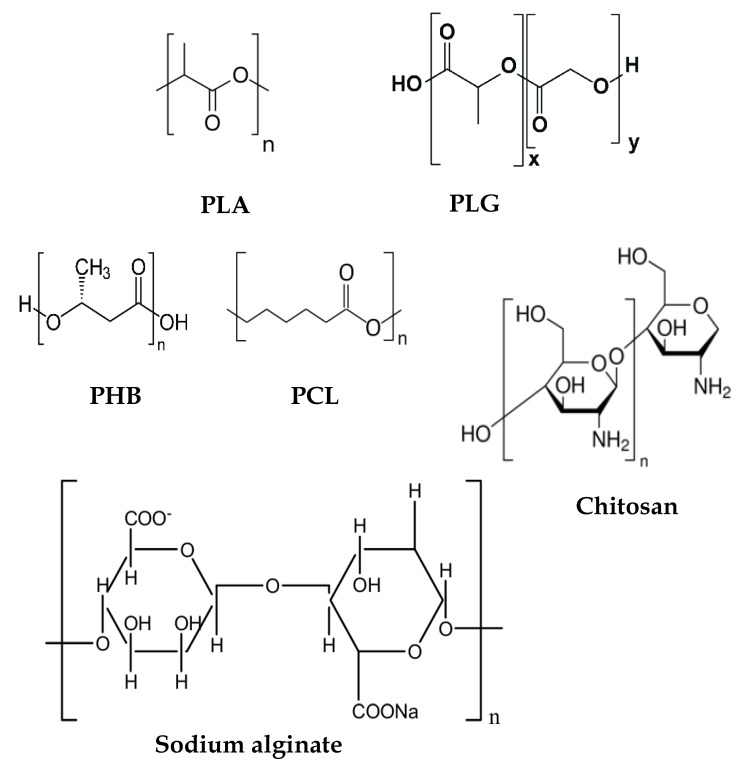
Chemical structure of some biocompatible polymers such as polylactic acid (PLA), poly (lactic-co-glycolic acid) (PLGA), polyhydroxybutyrate (PHB), polycaprolactone (PCL), chitosan, and sodium alginate.

In a brief overview, biocompatible polymers interact with biological systems without exerting significant damage or toxicity, displaying characteristics like biodegradability and/or biosorption. Their broad utility has been reported in different areas of medicine including orthopedics, tissue engineering, and drug and vaccine delivery; eventually, these materials also display intrinsic biological activity [192,193,194,216]. Medical devices or drug delivery systems based on biocompatible polymers have been approved by the Food and Drug Administration (FDA) [192,193,194,216]. Biocompatible polymers can be natural (e.g., chitosan, alginate, cellulose) or synthetic (e.g., polylactic acid, poly (lactic-co-glycolic acid)); some excellent reviews on biocompatible polymers are available [217,218]. One of the most promising delivery strategies for activating the immune system is the controlled release of antigens from a biodegradable polymeric matrix, the sustained liberation of antigens may induce strong immune responses [216,217]. Many biocompatible polymers have often been used as vaccine adjuvants such as the biocompatible and biodegradable poly (lactic acid) (PLA), poly (lactic-co-glycolic acid) (PLGA), and chitosan [216,219,220]. Figure 11 shows the chemical structures of some biocompatible polymers used for vaccine delivery.

As a first example of combination of cationic polymer and biocompatible polymer, assemblies of PEI and PLGA have often been used in vaccine delivery systems to carry antigens; they enhanced the antigen-specific antibody titer, lymphocyte proliferation, antigen internalization by phagocytic cells, and Th-1 typical cytokines production [136,221,222]. Biocompatible PLGA NPs were coated with three different cationic polymers, PEI, chitosan, or ε-Poly-l-lysine (PLL), aiming at improving the antigen loading capacity of PLGA NPs and evaluating the immune response against OVA as model antigen. PLGA-PEI/OVA NPs displayed the highest OVA loading capacity and colloidal stability, improved lymphocyte proliferation, CD4/CD8 ratio, increased the production of OVA-specific IgG1 and IgG2a antibodies and the secretion of TNF-α, IFN-gamma, IL-4, and IL-6 [136]. Similarly, the cationic PEI-coated PLGA NPs encapsulating the immunopotentiator *Angelica sinensis* polysaccharide (ASP) significantly activated macrophages, induced the expression of MHC II and CD86 molecules and the production of IL-1β and IL-12p70 cytokines. The adsorption of the porcine circovirus type 2 (PCV2) antigen onto NPs improved the macrophages antigen internalization. Immunized mice significantly improved the production of PCV2-specific IgG and responded with a mixed Th-1/Th-2 biased immune response [221].

Microparticles of PLGA coated with branched or linear PEI were suitable for delivering DNA vaccines demonstrating low cytotoxicity and improvement of APCs internalization [223,224]. In an interesting approach, PEI/DNA complexes were entrapped into PLGA microspheres warranting protection against DNA degradation and efficient uptake by APCs in comparison to the one of naked DNA. The humoral response elicited by PLGA/PEI/DNA against an DNA-HIV-1 antigen was from two to three times higher than one elicited by naked DNA; the cytolytic T lymphocyte at low doses of the antigen was also improved by the complex [224].

Other pairs of polymers used for assembling polymer–polymer hybrid NPs were PLGA/chitosan or PLGA/chitosan-derivatives, these hybrid NPs also improved the uptake rates of antigen by APCs and enhanced the elicited immune response in vivo [191,225,226]. By a modified double emulsion solvent evaporation process, three different NPs based on PLGA were synthesized, chitosan-coated PLGA, glycol-chitosan-coated PLGA, and PLGA NPs, all of them with a diameter smaller than 200 nm and positive zeta potentials; the NPs were used as adjuvants for assessing their capability to induce an enhance immune response. After immunization, glycol-chitosan/PLGA NPs elicited the higher mucosal and systemic response when administered with hepatitis B surface antigen, associated due to in vitro mucoadhesion. Both coated NPs experimented on had less mucosal clearance than PLGA uncoated NPs, resulting in improvement of the immune response [226]; permanence of the vaccine at the mucosa improved the obtained response [226,227,228]. Slow antigen-releasing N-trimethyl-chitosan-coated-PLGA/OVA NPs with permanent cationic charges yielded high IgA production [229].

Assemblies of PLA with cationic polymer physical properties and immunoadjuvant activity were described [230,231]. Polycation-coated PLA NPs increased the expression of MHC and co-stimulatory molecules, enhanced cytokine production, activated and induced maturation of APC, and improved antibody titer [147,227]. PLA microspheres coated with PEI or chitosan or chitosan chloride significantly increased the loading antigen capacity according to the increment of surface charge; coating PLA microspheres with polycations also augmented the macrophages uptake rates of the adsorbed hepatitis B surface antigen, the expression of CD86, MHC I, MHC II, and the secretion of IL-1β, IL-6, TNF-α, and IL-12. Intraperitoneal administration of cationic microspheres carrying the antigen improved the production of antibodies as compared with aluminum-based adjuvant and free antigen; besides, the cationic microspheres elicited a Th-1 biased showing the assemblies adequacy for performing antiviral, antimalaria, and antitumor vaccines [227].

**Figure 12:**
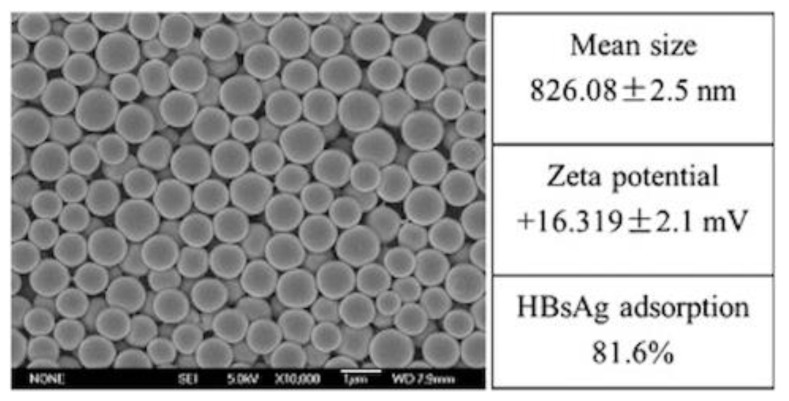
Scanning electron micrographs and dynamic light scattering Zetasizer analysis were used to characterize PLA microparticles (MP) modified by cationic chitosan chloride surface coating. Antigen adsorption was calculated by comparing the amount of antigen in supernatants after centrifugation with total input antigen (protein concentration was analytically determined). The load of HBsAg/particles was 3.2 (μg/mg). Reprinted from [147] with permission from Elsevier, Copyright 2014.

Chen and coworkers tested chitosan-decorated PLA microspheres carrying hepatitis surface antigen administered via commonly used parenteral administration routes and obtained robust immune responses such as maturation of DCs with overexpression of CD40, CD80, and IL-12 in contrast with the absence of these responses when aluminum-based adjuvant was used; in vivo tests showed increased IgG production induced by both the MPs and the alum-based adjuvants; only the MPs administered by intramuscular route could induce high IgG2a titer, Th-1 cytokines (IL-2, IL-12, and IFN-γ) and Th-2 cytokine IL-4 secretions, demonstrating that PLA-coated NPs improve the cellular and humoral response [147]. Figure 12 shows a scanning electron micrograph of PLA microparticles coated by chitosan chloride and gives details on mean diameter, zeta-potential, and antigen loading [147].

Alginate and chitosan are two versatile biopolymers widely used in drug delivery systems, as they are oppositely charged and it is relatively simple to obtain hybrid systems from them. Taking advantage of the physical–chemical properties of those polymers, one can obtain interesting systems for carrying a myriad of antigens like protein, DNA, or RNA antigen; for instance, it is possible to encapsulate the cargo into the hybrid assembly or to obtain surfaces positively or negatively charged for adsorbing antigens. Regarding the adjuvant properties, one of the main limitations of oral vaccines is that antigens suffer degradation into the gastric cavity losing their native structures and antigenicity; using alginate and chitosan, it is possible to avoid this phenomenon [232,233,234]. In an interesting work where hybrid NPs from chitosan and alginate were synthesized for vaccine delivery, it was shown that alginate coating antigen/chitosan complex is a good strategy for avoiding antigen degradation from gastric environment. The system showed a pattern of controlled release and low in vitro toxicity; furthermore, alginate/chitosan hybrid NPs elicited systemic and mucosal immune response exhibiting the highest titer of antibody as compared with the controls [234]. However, alginate/chitosan hybrid anionic NPs failed in inducing a Th1 response when the NPs and a hepatitis B virus antigen were administered by the subcutaneous route in a mouse model [235].

Alginate and PLL were assembled as NPs by ionotropic complexation method. The NPs displayed a diameter between 130 and 850 nm, a negative zeta-potential, and a sustained-release behavior regarding the encapsulated BSA. Additionally, the system showed low cytotoxicity and significant increment of BSA internalization in vitro [236].

Hybrid NPs from combinations of biocompatible and cationic polymers have been less studied as antigen carriers. For example, hybrid systems between PEI and alginate are little explored in the context of vaccine delivery, although there is evidence that the two polymers form complexes by electrostatic interactions [237]; combinations of PEI with alginate reduce significantly the toxicity of the polycation favoring the degradation of PEI [238]. Alginate/PEI hybrid systems formed nanogels able to incorporate and deliver antigens enhancing both cellular and humoral response. Nanogels facilitated antigen uptake by mouse bone-marrow DCs; promoted intracellular antigen degradation and cytosolic release, and increased antigen presentation via MHC I and II, vaccine-induced antibody production, and CD8+ T cell-mediated tumor cell lysis, suggesting that the nanogels are potent immunoadjuvants [239].

PEI-coated PMMA NPs synthesized by emulsion polymerization combined well with DNA and are a suitable alternative for gene delivery, showing low cytotoxicity and high transfection capacity [240,241]; however, these works did not report evidence of the immunoadjuvant activity of those assemblies.

**Figure 13:**
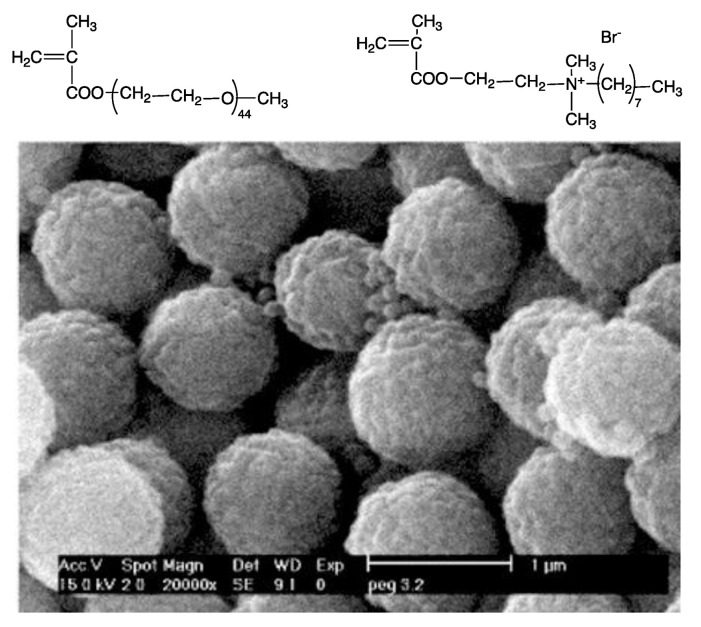
Scanning electron micrograph of PMMA core–PEG shell microparticles where the hydrophilic non-ionic co-monomer poly (ethylene glycol) methyl ether methacrylate and the cationic co-monomer 2-(dimethyloctyl) ammonium ethyl methacrylate bromine were used in the emulsion polymerization reaction. Reprinted from [242] with permission from Elsevier, Copyright 2011.

Using emulsion polymerization of the two co-monomers, interesting functionalities were reunited in the same polymeric particle: the cationic moiety represented by the quaternary ammonium nitrogen of comonomer 1 and the hydrophilic poly (ethyleneglycol) chains of comonomer 2; thereby poly (methylmethacrylate) (PMMA) was covalently modified to yield core–shell cationic nanoparticles that enhanced cellular responses induced by HIV-1 Tat DNA vaccination. These biocompatible core–shell cationic nanoparticles, composed of an inner hard core of poly (methylmethacrylate) (PMMA) and a hydrophilic tentacular shell bearing positively charged groups and poly (ethyleneglycol) chains covalently bound to the core electrostatically and reversibly adsorbed DNA, efficiently delivered it intracellularly and were not toxic in vitro or in mice [242]. Furthermore, two intramuscular (i.m.) immunizations (4 weeks apart) with a very low dose (1 microgram) of the plasmid PCV-tat delivered by these nanoparticles followed by one or two protein boosts induced significant antigen-specific humoral and cellular responses and greatly increased Th1-type T cell responses and CTLs against HIV-1 Tat [242]. Along similar lines, PEG was covalently bound to the cationic polymer poly (2-aminoethyl methacrylate hydrochloride) in order to improve the stability of nanstructures in vivo [243]. Figure 13 shows the core–shell microparticles obtained by Castaldello and coworkers, 2006, incorporating both functionalities, cationic charge and the stability, in aqueous medium imparted by PEG [242].

**Figure 14:**
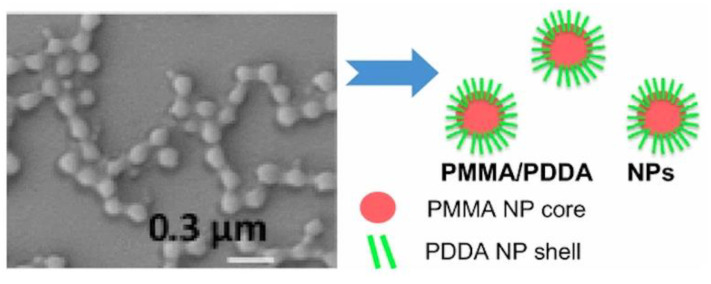
Scanning electron micrograph of PMMA/PDDA NPs and schematic representation of the PMMA/PDDA NPs structure with their core–shell nanostructure that occurs at low ionic strength. Reproduced from reference [160].

Non-covalent nanoparticle (NP) assemblies of PMMA, a synthetic, non-charged, and biocompatible polymer, and PDDA polycation have been described earlier by our group since the first report on the good compatibility and miscibility of PDDA with PMMA evaluated from microbicidal PMMA/PDDA NPs [160,161,162,163,210,211,212,213,214]. The synthesis of PMMA NPs by emulsion polymerization of MMA in the presence of PDDA yielded cationic, homodisperse, and stable NPs [160,212], which are presently being evaluated in our group for possible applications as immunoadjuvants. In summary, it is possible to take advantage of the biocompatibility of PMMA and the cationic character of PDDA to investigate novel applications for these assemblies obtained in absence of any covalent linkage between the polymers. In addition, our group recently synthesized and characterized hybrid NPs from PMMA, PDDA, and surfactants that could be excellent alternatives for carrying antigen due to the combination of the biocompatibility of PMMA with the adjuvant properties of the cationic polymer PDDA and/or the cationic lipid DODAB [65,161,162]. Figure 14 shows the core–shell PMMA/PDDA NPs and a schematic picture of their nanostructure with PMMA in the core and PDDA non-covalently bound to PMMA, forming a surrounding shell [214]. One should notice that the hydrophobic PMMA is in the core and the cationic and hydrophilic PDDA is in the shell; upon increasing the ionic strength, the shell can collapse due to the screening of the PDDA charges [19,20,21,22,23,24,25,26,27,28,29,30,31,32,33,34,35,36,37,38,39,40,41,42,43,44,45,46,47,48,49,50,51,52,53,54,55,56,57,58,59,60,61,62,63,64,65,66,67,68,69,70,71].

**Figure 15:**
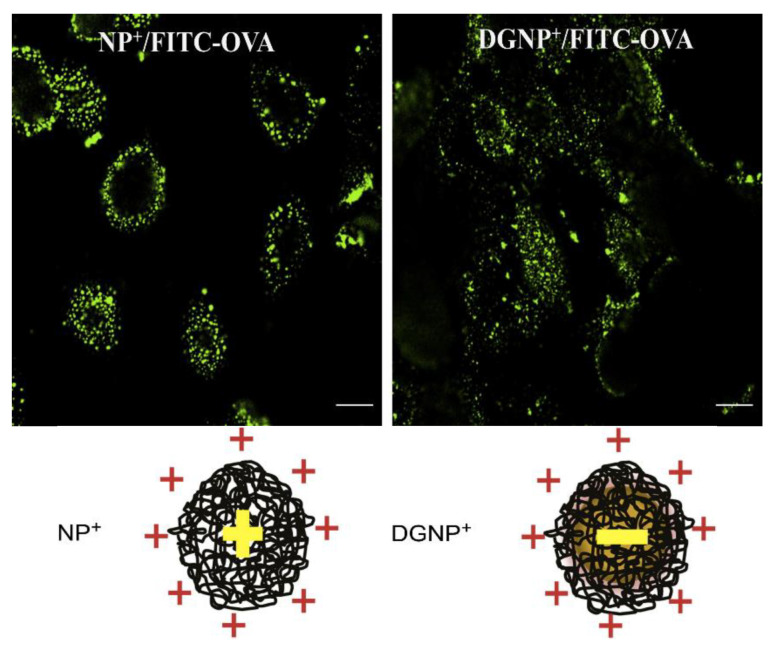
Comparison of ovalbumin (OVA) intracellular delivery by maltodextrin porous nanoparticles with outer and inner cationic charges (NP+) or outer cationic/inner anionic charges (DGNP+) using confocal microscopy to visualize OVA labeled with fluorescent markers (FITC-OVA). NP+ and DGNP+ were loaded with FITC–OVA and incubated for different periods with 16HBE cells. After 30 min incubation, cells were washed with PBS and fixed with 4% PAF. Intracellular FITC–OVA was visualized by confocal microscopy. Scale bar = 10 μm. Adapted from [244] with permission from Elsevier, Copyright 2012.

Antigen endosomal escape has been observed for protein loaded in cationic and porous maltodextrin NPs [244]. Figure 15 illustrates the endosomal escape or permanence of two types of cationic maltodextrin NPs, those modified with anionic lipids in their cores (named DGNP^+^) and those with cationic cores (named NP^+^). The OVA/NPs intracellular traffic was determined from fluorescence-labeled OVA. Both types of NPs efficiently delivered OVA to cells; however, whereas those with the anionic inner core delivered OVA to the cytosol and endoplasmic reticulum, those with the cationic core were less able to release OVA in the cytosol and delivered it to the endosomes. One should notice the punctuated distribution pattern of OVA delivered by NP+ into cells, typical of endosomal localization and the diffused OVA localization throughout the cytoplasm when OVA was delivered with DGNP+ NPs. Both NPs increased intracellular proteolysis of OVA; however, DGNP(+) facilitated OVA escape from endosomes [244].

**Figure 16:**
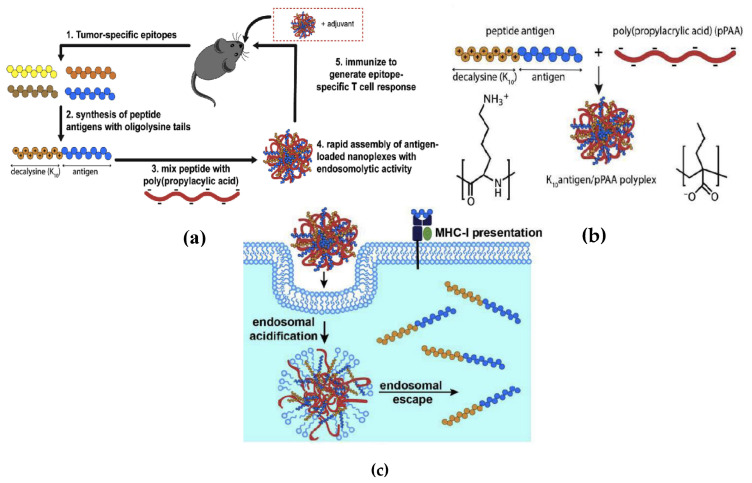
(**a**) Poly (propylacrylic acid)-cationic peptide nanosized assemblies for enhancing antigen endosomal escape and MHC-I antigen presentation. (**b**) Assembly of antigen-loaded nanoplexes via simple and rapid mixing of decalysine-modified antigenic peptides and pPAA, which generates electrostatically stabilized nanoparticles. (**c**) Schematic representation of nanoplexes promoting cytosolic antigen delivery via endosomal escape, resulting in enhanced levels of antigen presentation on class I major histocompatibility complex (MHC-I). Reprinted from [245] with permission from Elsevier, Copyright 2018.

Cancer vaccines targeting patient-specific tumor neo-antigens have recently emerged as a promising component of the immunotherapeutic treatment against the disease [245,246]. However, neo-antigenic peptides typically elicit weak CD8^+^ T cell responses, so there is a need for universally applicable vaccine delivery strategies to enhance the immunogenicity of these peptides. Ideally, such vaccines could also be rapidly fabricated using chemically synthesized peptide antigens customized to an individual patient. An interesting approach for implementing the combination between biocompatible polymer and cationic polymer was achieved with the biocompatible propyl-acrylic acid and cationized antigen from covalently binding decalysine peptide to the antigen and then assembling this with the biocompatible polymer via electrostatic assembly; these NPs promoted cytosolic delivery of the antigen and MHC I presentation [245,247]. In addition, poly (acrylic acid) derivatives as mucoadhesive polymers might increase the epithelial permeability for carried antigens or drugs after easily crossing the mucus layer of the mucosae. The attachment to the mucus layer could be achieved by exclusively non-covalent bonds such as ionic interactions, hydrogen bonds, and van der Waal’s forces, leading also to a certain extent to opening of the tight junctions [248]. Figure 16 shows a schematic representation of the electrostatically stabilized NPs assembled by mixing decalysine-modified antigenic peptides and poly (propyl acrylic acid) (pPAA) poly-anion. NPs increased and prolonged antigen uptake and presentation on MHC-I (major histocompatibility complex class I) molecules expressed by dendritic cells with activation of CD8^+^ T cells. The suitable intranasal immunization route inhibited formation of lung metastases in a murine melanoma model; further addition of the adjuvant α-galactosylceramide (α-GalCer) stimulated robust CD8^+^ T cell responses, significantly increasing survival time in mice with established melanoma tumors [245]. On Figure 16a, poly (propylacrylic acid)-cationic peptide nanosized assemblies aiming at enhancing antigen endosomal escape and MHC-I antigen presentation are prepared. On Figure 16b, one should notice the simple simple and rapid mixing of decalysine-modified antigenic peptides with poly (propylacrylic acid) (pPAA) forming antigen-loaded nanoplexes, which are electrostatically stabilized nanoparticles. On Figure 16c the scheme shows the nanoplexes promoting cytosolic antigen delivery via endosomal escape, resulting in enhanced levels of antigen presentation on class I major histocompatibility complex (MHC-I).

Inside the endosome, PLGA erosion with release of the glycolic or lactic acids further reduced the pH, improving the antigen degradation and presentation through MHC-II [249,250]. Oral administration of antigens may lead to their uptake by microfold cells (M cells) in Peyer’s patches of intestine to initiate protective immunity against infections, but limitations such as the lack of specificity of proteins toward M cells and degradation of proteins in the harsh environment of gastrointestinal (GI) tract had to be overcome. Mucoadhesive vehicle of thiolated eudragit (TE) microparticles transported an M cell-targeting peptide-fused model protein antigen [251]. Thereby, oral delivery of TE microparticulate antigens exhibited high transcytosis of antigens through M cells resulting in strong protective sIgA as well as systemic IgG antibody responses. The delivery system not only induced CD4+ T cell immune responses but also generated strong CD8+ T cell responses with enhanced production of IFN-γ in spleen [251]. Along similar lines, the mucoadhesive, cationic, and biocompatible chitosan in combination with poly-ε-caprolactone yielded NPs effective in immunization against influenza [252].

In conclusion, hybrid assemblies of polycation and biocompatible polymer have the advantages of reducing the toxicity of the cationic polymer and adding the interesting properties of muco-adhesiveness so important for mucosal vaccines.

## 5. Cationic Assemblies of Lipid–Polymer and Polymer–Lipid

Assemblies obtained from combining lipids and polymers gather the benefits of lipids’ amphiphilic nature, possibility of high organizational level in the lipid-based structures, and the mechanical and resistance advantages offered by polymers; blending these materials, it is possible to overcome major limitations like degradation of bioactive incorporated principles, short circulation time of drugs and vaccines, and lack of controlled release [253,254]. Diverse types of arrangement between lipids and polymers have been described; in general, these hybrid assemblies could be classified as lipid–polymer or polymer–lipid, depending on the position of each material in the hybrid structure; either the polymer or the lipid is in the inner part the nanostructure. In addition, polymer and lipid may mix, driven by intermolecular interactions, so that the lipid may become embedded in the polymer matrix displaying good compatibility with the polymer [210,212,213,253,254].

Lipids and polymers can assemble as hybrid materials driven by attractive, non-covalent, and weak but frequent multipoint interactions. For example, lipid deposition from charged bilayers onto oppositely charged spherical solid cores such as polymeric nanoparticles [37,205,255], silica [208,256,257,258], or hydrophobic drug aggregates [38,259] led to interesting and bioactive hybrid nanoparticles. The so-called biomimetic, lipid–polymer, or polymer–lipid NPs have been finding applications in biomolecular recognition [260], drug delivery [259,261,262,263], vaccine design [9,10,16,18,21,25,28,46,163], and antimicrobial chemotherapy [131,139,140,141,160,163,207,211,212,263,264,265,266,267,268,269,270,271].

**Figure 17:**
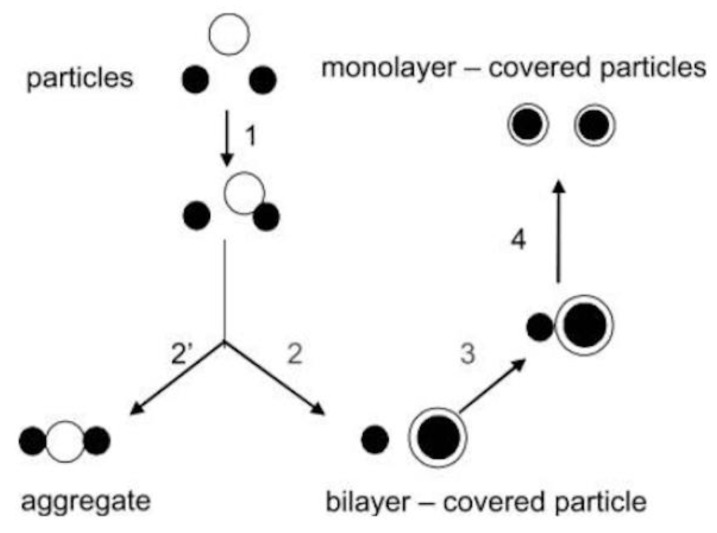
The interaction between one bilayer vesicle and two particles. In the first step (step **1**), electrostatic and/or van der Waals and/or hydrophobic attraction leads to aggregation of a vesicle and a particle. These same interaction forces may disrupt the vesicle bilayer and promote bilayer adsorption onto the microsphere (step **2**) and/or further aggregation with the other microsphere (step 2′). The adsorbed bilayer may attract the second microsphere (step **3**). The hydrophobic interaction between an eventually hydrophobic surface and the hydrocarbon chains in the bilayer may completely destroy the bilayer structure, flip-flopping the hydrocarbon chains onto the particle surface and generating a monolayer coverage on each microsphere (step **4**). Adapted from [206] with permission from Elsevier, Copyright 1999.

The principal forces driving bilayer deposition onto hydrophobic or hydrophilic nanoparticles or surfaces from bilayer vesicles or bilayer fragments (BF) are the electrostatic attraction, van der Waals attraction, and/or the hydrophobic effect [37,205,255,256,258]. Medium composition, pH, and the ratio between the surface areas of the particles (Aparticles) and bilayers (Abilayers) also play a crucial role in achieving the optimal bilayer deposition on the particles: this ratio should be around 1 for complete coverage of all particles with bilayers [38,208,259,260,261,262,263]. The amount of added lipid must be sufficient to surround all particles in the dispersion with one bilayer; otherwise, poor colloidal stability may result with formation of aggregates. Figure 17 illustrates some possible interactions between one bilayer vesicle and two particles, vesicle, and particles with similar sizes [206].

When lipid bilayers are the starting nanostructure, their physical state is an important factor determining lipid–particle interactions; bilayer vesicles in the rigid gel state do not disrupt upon adhesion onto solid particles, so bilayer coverage does not take place [272]. For avoiding this difficulty, either the lipid has to be changed to a lipid able to form bilayers in the liquid-crystalline, more fluid state at room temperature, or flat and open bilayer fragments of the charged lipid (BF) has to be used. BFs not only can adsorb onto solid particles but also can independently function as scaffolds for deposition of a variety of bioactive molecules such as peptides [207,265,267,268], drugs [131,207,259,263,264,269], antimicrobial polymers [139,141,163,266,273], proteins [21,25,28,40,46,140,141,163,260,264], oligonucleotides [16,46], or nucleic acids [41,42]. Adhesion of a DODAB vesicle layer onto the rough and highly hydrated surface of cells was electrostatically driven; cationic closed vesicles of DODAB at low ionic strength surrounded bacterial cells as a vesicle layer [274]; the absence of DODAB vesicle disruption upon interaction with the bacteria was depicted from absence of (14C)-sucrose leakage from the large vesicles in experiments where this marker was used to label the inner water compartment of the vesicles [275]. Given the quaternary ammonium moiety of the DODAB molecule, its antimicrobial effect was systematically evaluated and its differential cytotoxicity was reviewed [38].

**Figure 18:**
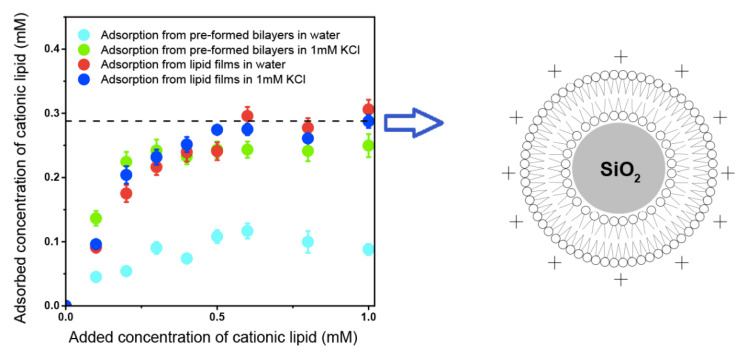
Adsorption isotherms for DODAB from films or pre-formed bilayer fragments (BF) onto silica (2 mg/mL). The dashed line at 0.288 mM DODAB represents the theoretical concentration corresponding to bilayer adsorption. Reproduced from [209].

Charged BFs are easily obtained from vesicles by ultrasonic disruption and have been used for the production of a variety of lipid-based biomimetic particles [37,38,39,41]. Deconstruction of the bilayer by solubilizing cationic lipid and drug or biocompatible polymer in a common solvent has also been a useful approach; this successfully allowed the obtaining of hybrid nanoparticles of hydrophobic drug/cationic lipid/ethanol in water dispersion surrounded or not by the biocompatible carboxy methyl cellulose biopolymer [276]. Another successful strategy was the optimization of single bilayer deposition onto silica nanoparticles from lipid films [209]. In order to ascertain whether bilayer coverage indeed took place, size distribution, polydispersity, and zeta-potential are often determined from dynamic light scattering (DLS) techniques [277] complemented by morphology evaluation from advanced electron microscopy techniques [131,160,212] and quantitative methods for obtaining adsorption isotherms [209]. Figure 18 shows some adsorption isotherms of the cationic lipid DODAB from DODAB films or from pre-formed bilayers onto silica particles where maximal adsorption values yielded an adsorbed amount consistent with bilayer deposition on silica for depositions from DODAB films [209].

If the lipid bilayers and the particles are oppositely charged, poor colloidal stability occurs at a critical lipid concentration where the size is maximum and the zeta-potential is zero [10,18,205,208,259,263]. Above this critical lipid concentration, the particles exhibit zeta-potentials that are similar to that of the charged bilayer and recover colloidal stability [10,18,205,259]. Aggregates of hydrophobic drugs dispersed in water have also been treated as particles and coated with lipids at high drug-to-lipid molar ratios [140,259,263]. More recently, protocols have evolved to employ bilayer disassembly and biopolymers for stabilization of very hydrophobic drugs such as indomethacin [163,276].

**Figure 19:**
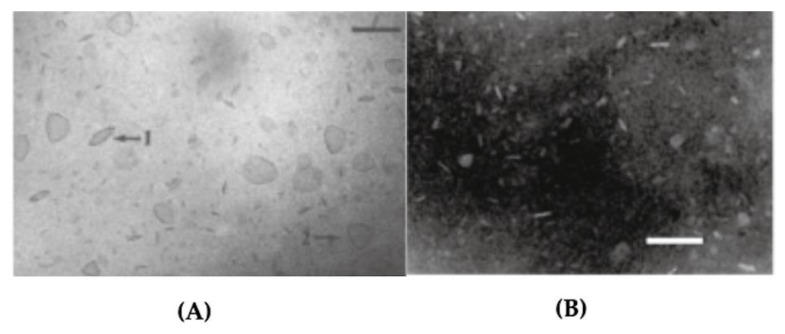
Lipid bilayer fragments of cationic and anionic synthetic lipids. (**A**) Bilayer fragments of dioctadecyldimethylammonium bromide (DODAB) visualized by cryo-transmission electron micrograph. Adapted from [278] with permission from 1995 American Chemical Society. (**B**) Bilayer fragments of sodium dihexadecylphosphate (DHP) visualized by transmission electron micrograph after negatively staining the sample. Disks were observed edge-on or face-on. Bars denote 100 nm Adapted from [279] with permission from 1991 American Chemical Society [278,279].

BFs combine well with several antigens and are available as cationic (made of DODAB) or anionic nanostructures (made of the anionic and synthetic lipid sodium dihexadecylphosphate or DHP), allowing combinations with both positively and negatively charged antigens. Transmission electron micrographs of DODAB BF [278] and DHP BF [279] are on Figure 19A,B, respectively.

DODAB BFs loaded or unloaded with antibiotics were covered consecutively by a carboxymethylcellulose (CMC) and a poly (diallyl dimethyl ammonium chloride) (PDDA) layer [131,141] being efficiently captured by macrophages to deliver their antibiotic cargo against difficult intracellular pathogens, as are the mycobacteria [270]. The activity of DODAB BF/CMC or DODAB BF/CMC/PDDA in combination with antigens still requires further research.

**Figure 20:**
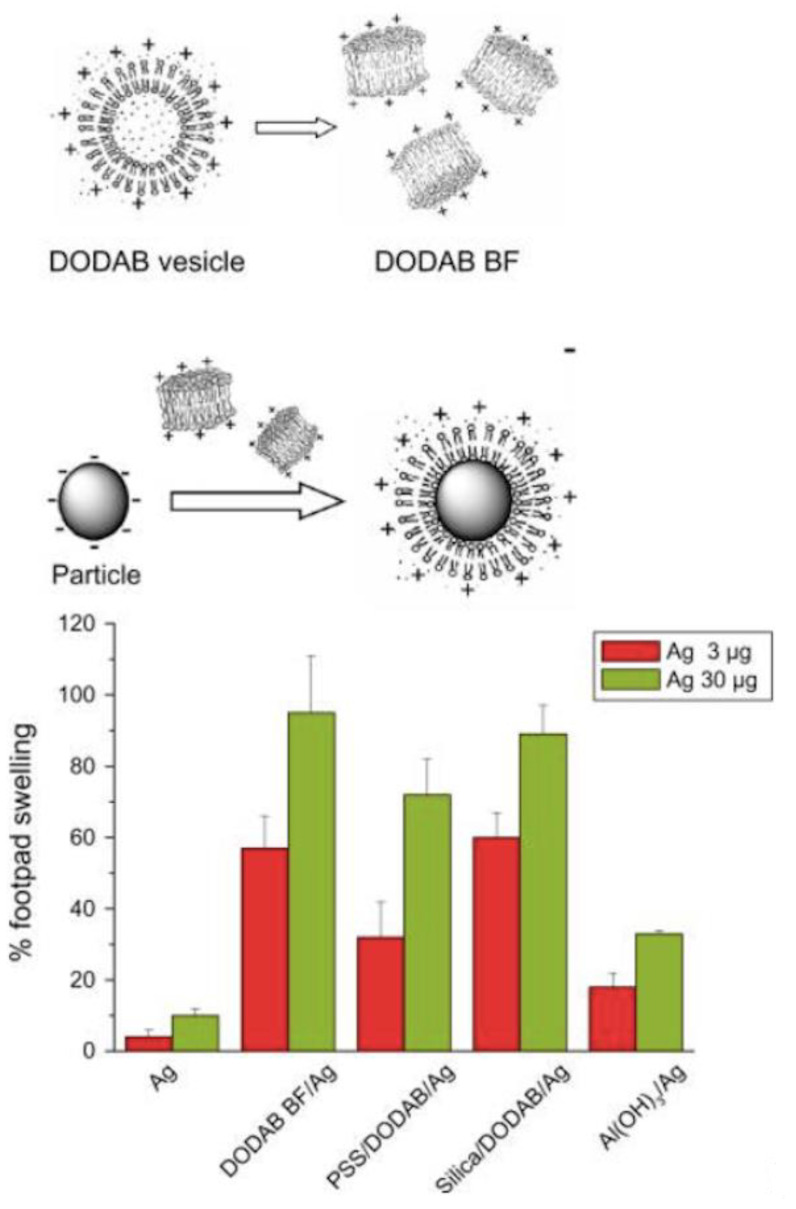
Schematic representation of nanometric dioctadecyldimethylammonium bromide (DODAB)-based adjuvants inducing delayed-type hypersensitivity (DTH) in mice as compared to alum. The same antigen (Ag) carried by each adjuvant was used for immunization. Ag was carried by DODAB BF at 0.1 mM DODAB (DODAB BF/Ag) or by polystyrene sulfate nanoparticles (PSS)/DODAB or by silica/DODAB particles at 0.01 or 0.05 mM DODAB (PSS/DODAB/Ag or silica/DODAB/Ag), respectively, or by alum (Al(OH)_3_/Ag). After immunization, elicitation of the swelling response was done by injecting Ag alone in the mice footpad so that % footpad swelling was measured in comparison to alum [9,10,18]. At the DODAB doses employed, DODAB cytotoxicity was absent against mammalian cells in culture. Adapted from [9,10] with permission from Elsevier, Copyright 2007 and Copyright 2009 and reference [18].

The differential cytotoxicity of DODAB, its dose-dependent toxicity, and its ability to induce delayed-type hypersensitivity (DTH) in vivo, a marker for cell-mediated immune responses, pointed out the feasibility of using DODAB as an efficient immunoadjuvant mainly for veterinary uses but also in humans in a few instances [9,13,52,57,280,281,282]. Supramolecular assemblies of DODAB BF by themselves or after interaction with supporting particles were also combined with three different model antigens in separate and tested as immunoadjuvants [9]. DODAB-based immunoadjuvants carrying antigens at reduced DODAB dose (0.01–0.1 mM) induced superior DTH responses in mice in comparison to alum. Thus, the cationic immunoadjuvant was either reduced to a single-component, nanosized system—DODAB BF—or was a dispersion of cationic nanoparticles with controllable nature and size as obtained after covering silica or polystyrene sulfate latex (PSS) with a cationic DODAB bilayer. DODAB BF interacted with proteins via both the hydrophobic effect and the electrostatic attraction at low ionic strength. DODAB-based adjuvants exhibited good colloid stability while complexed with the antigens, complete absence of toxicity in mice (i.e., local or general reactions), and a remarkable induction of Th1 immune response at reduced doses of cationic and toxic DODAB lipid. DODAB vesicle disruption by probe sonication at low ionic strength (0.1–5.0 mM monovalent salt) produced DODAB BF which remained electrostatically stabilized in dispersion by the electrostatic repulsion in between fragments. DODAB BF also interacted with oppositely charged particles such as silica or polystyrene sulfate (PSS) latex to produce the cationic particulates. Figure 20 shows some hybrid and cationic immunoadjuvants based on reduced DODAB doses and their compared DTH response [9,10,18].

An interesting approach was described in order to apply immunotherapy associated with chemotherapy against *Leishmania* infection: solid lipid NPs using soya phosphatidyl choline and stearic acid were prepared by solvent emulsification-evaporation method followed by ultrasonication. These solid NPs were loaded with amphotericin B before coating with chitosan and displayed diameters lower than 220 nm and positive zeta-potentials showing low hemolytic activity and antileishmanial activity higher than the one for commercial AmBisome or Fungizone. The NPs were taken up by J774A.1 macrophages, which activated the production of TNF-α and IL-12; importantly, cytotoxicity experiments in vitro and acute toxicity experiments in mice evidenced the safety of the formulation in comparison to marketed formulations [283].

Cationic and porous NPs from maltodextrin and the anionic lipid 1,2-dipalmitoyl-sn-glycero-3-phosphatidylglycerol (DPPG) were synthesized to evaluate their interaction with the nose mucosa; these NPs, combined with OVA displayed low toxicity, were efficiently taken up by airway epithelial cells and significantly improved the OVA delivery to the cells, increasing the permanence time of OVA at the nose mucosa to at least 6 h, in contrast to unformulated OVA, which remained 1.5 h only at the mucosa [284].

DODAB coating the biocompatible PLGA yielded cationic nanoparticles that elicited Th1 and Th17 responses [284,285]. Cationic NPs of PLGA, DODAB, and TDB in a nasal vaccine displayed nanometric sizes and high and positive zeta-potentials while carrying the outer-membrane protein (MOMP) antigen of *Chlamydia trachomatis,* inducing high titers of IgG2a, IFN-ɣ, and IL-17a [286].

Similarly, PLGA and DODAB were combined to yield cationic NPs for delivering a *Mycobacterium tuberculosis* nasal vaccine; PLGA/DODAB NPs showed uniform size, spherical shape, and smooth surface. PLGA/DODAB NPs loaded with HspX/EsxS antigen (a recombinant fused protein of *M. tuberculosis*) and with monophosphoryl lipid A (MPLA) increased the secretion of IFN-γ and IL-17 and enhanced the antibody titers of IgA, IgG1, and IgG2a [285]. In a different approach, cationic liposomes obtained from DOTAP, hyaluronic acid (HA), and PEG achieved a good immune response after intranasal vaccination with a recombinant antigen from *M. tuberculosis*; there was improved colloidal stability and prolonged antigen release. Besides, the typical cytotoxicity exerted by DOTAP was reduced 20-fold. DOTAP/HA/PEG NPs carrying OVA and MPLA promoted DCs maturation and up-regulation of co-stimulatory markers such as CD40, CD86, and MHC-II. Mice vaccinated with DOTAP/HA/PEG NPs carrying OVA and MPLA via intranasal route generated robust OVA-specific CD8(+) T cell and humoral responses; in addition, the intranasal inoculation of DOTAP/HA/PEG NPs co-loaded with MPLA and F1-V, a fused antigen from *Yersinia pestis*, induced a potent and long-lasting antibody production, evidencing that these hybrid liposome/polymer NPs are appropriate as a mucosal adjuvant [287].

PLGA/DC-Chol core/shell hybrid NPs synthesized by a double emulsion solvent evaporation method were tested as an adjuvant with the peculiarity that the antigen, OVA, was attached to the NPs in three different ways: inside the NPs (OVA in), adsorbed on the NPs (OVA ad), or both ways (OVA in/ad). After the internalization by DCs, FITC-OVA traffic from fluorescence microscopy showed that OVA ad and free OVA remained in the lysosomes, whereas OVA in or in/ad escaped from the endo-lysosome favoring cross-presentation. In vivo experiments showed that OVA in/ad provided not only adequate initial antigen exposure but also long-term antigen persistence at the injection inside. OVA in and OVA in/ad elicited significantly higher antigen-specific immune response than OVA ad [288].

Summarizing this topic, cationic lipids are very versatile and can either carry antigens by themselves via BF or impart positive charges to a variety of polymeric NPs under good control of their usual toxicity. They have the advantage of eliciting a potent cellular immune response in many instances. The adjuvant properties of the hybrid NPs have been often reviewed [21,25,34].

## 6. Conclusions

Cationic nanostructures such as the lipid-covered particles, the bilayer fragments, the cationic polymers, and the hybrid nanostructures of biocompatible and cationic polymers or lipids are interesting carriers with sizes below 100 nm. Their size directs them to the lymphatic vessels and antigen-presenting cells in the lymph nodes, and their positive charge allows efficient combination with important biomolecules such as peptides, proteins, nucleic acids, epitopes, and enhancers of the immune response.

The composition with lipids allows the inclusion of targeting in the microstructures that cannot directly reach the lymph nodes or that require special targeting to avoid degradation in the endosomes or to reach the cell nucleus.

The cationic nanostructures can protect the carried antigen for vaccine administration by the oral route and can increase the permanence time of the antigen/carrier assembly at the mucosae still enhancing the systemic immunity. The cationic nanostructures are particularly efficient in delivering antigens to APCs, allowing both antigen processing and presentation via MHC-II or processing and cross-presentation via MHC-I. The intracellular traffic depends on administration route and location of the antigen in the complex. The direct connection of the complex antigen/adjuvant to APCs in the lymph nodes avoids permanence at injection sites and local inflammatory reactions since they easily overcome the anatomical barriers due to their nanometric size. Their toxicity is easily controllable by using low concentrations of the cationic lipid or cationic polymer in the nanostructures.

There is a huge variety of cationic nanostructured materials available from nanomaterials science. However, most of them remain untested regarding their properties as immunoadjuvants or their intrinsic toxicity both in vitro and in vivo. This means that a huge area for biomedical research remains still unexplored and it is our hope that this review will be the trigger for further valuable research on vaccine design with novel cationic nanostructures.
